# Therapeutic Treatment Plan Optimization during the COVID-19 Pandemic: A Comprehensive Physicochemical Compatibility Study of Intensive Care Units Selected Drugs

**DOI:** 10.3390/pharmaceutics14030550

**Published:** 2022-02-28

**Authors:** Maria Gloria Tarantini, Stéphanie Ramos, Philippe-Henri Secrétan, Laura Guichard, Lamia Hassani, Agnès Bellanger, Julien Mayaux, Patrick Tilleul, Fadwa El Kouari, Hassane Sadou Yayé

**Affiliations:** 1 Department of Pharmacy, Groupe Hospitalier Pitié-Salpêtrière, Assistance Publique-Hôpitaux de Paris-Sorbonne Université, 47-83 Boulevard de l’Hôpital, 75013 Paris, France; maria.tarantini@edu.unito.it (M.G.T.); stephanie.ramos@aphp.fr (S.R.); laura.guichard@u-psud.fr (L.G.); lamia.hassani@aphp.fr (L.H.); agnes.bellanger@aphp.fr (A.B.); patrick.tilleul@aphp.fr (P.T.); fadwa.el-kouari@aphp.fr (F.E.K.); 2 Dipartimento di Scienza e Tecnologia del Farmaco, Università di Torino, Via Pietro Giuria, 11, 10125 Torino, Italy; 3 Matériaux et Santé, Université Paris-Saclay, 92296 Châtenay-Malabry, France; philippe-henri.secretan@universite-paris-saclay.fr; 4 Department of Pulmonology Intensive Care Medicine and Resuscitation, Groupe Hospitalier Pitié-Salpêtrière, Assistance Publique-Hôpitaux de Paris-Sorbonne Université, 47-83 Boulevard de l’Hôpital, 75013 Paris, France; julien.mayaux@aphp.fr; 5 Faculty of Pharmacy, University of Paris-Descartes, 4 Avenue de l’Observatoire, 75006 Paris, France

**Keywords:** SARS-CoV-2 pandemic, intensive care units, drugs mixtures (Sufentanil, Clonidine, Loxapine, Midazolam, and Ketamine), compatibility study, LC stability-indicating assay method, degradation kinetic

## Abstract

Background: The SARS-CoV-2 pandemic has resulted in a dramatic rise of the demand for medical devices and drugs. In this context, an important shortage of programmable syringe pumps, used to administrate different drugs in intensive care units, was seen. The opportunity of administrating combinations of five intensive care units selected drugs (Sufentanil, Clonidine, Loxapine, Midazolam, and Ketamine) was considered. Methods: The drug mixtures were studied in a pure form or diluted in NaCl 0.9% or G5%. Twenty-six possible combinations of the five drugs were produced in glass vials or polypropylene syringes and stored at 25 °C for 14 days. The LC method was implemented to study drugs combinations in the presence of the degradation products. The clearness and pH were also monitored. Results: All the 26 possible combinations displayed adequate physicochemical stability at 25 °C: at least 3 days and 7 days, respectively, for the dilution in 0.9% NaCl or glucose 5%, and the pure drug products mixtures. Conclusions: The study provided sufficient stability results, covering the medication administration period of at least three days. The combination of more than two drugs offers the advantage of minimizing the individual doses and reduces unwanted side-effects. Hence, this study opens up the possibility of combining the five drugs in one single syringe, which is useful especially under the current circumstances associated with an important shortage of programmable syringe pumps and pharmaceuticals.

## 1. Introduction

During the first wave of the 2019 SARS-CoV-2 pandemic that hit many countries worldwide, health systems were put under severe strain as hospitals experienced a significant influx of patients. The severity of clinical signs required that approximately 30% of patients remain in hospital and 5% be admitted to intensive care [1], leading to the overcrowding of intensive care units (three times the average number of patients in intensive care units at Pitié-Salpêtrière hospital of Paris) and a dramatic rise in the demand for medical devices and drugs. 

In this context, we faced a shortage of programmable syringe pumps used to administer different drugs in intensive care units, especially Sufentanil, Clonidine, Loxapine, Midazolam, and Ketamine, on which the study is focused. To face this shortage, the opportunity of administrating two or more combined drugs was considered, in accordance with medical staff. 

The intrinsic stability of some of these drugs alone has already been described in the literature, but rarely in combination with other drugs, and drug–drug compatibility studies were often limited to two drug products. 

Midazolam hydrochloride is a short-acting, injectable benzodiazepine commonly used in hospitals for preoperative or conscious sedation, general anxiolysis, and induction of general anesthesia. It is administered in intensive care units as a continuous i.v. infusion to achieve long-term sedation. The drug product appears to be poorly affected by light and temperature. However, it is described to undergo hydrolysis degradation [2,3].

Ketamine is a non-barbiturate dissociative anesthetic, used either alone for short-term medical procedures (rapid sequence intubation, short-term procedural sedation, etc.), or in combination with other medications as a pre-anesthetic. As well as Midazolam, Ketamine solution diluted with sterile water for injection showed remarkable chemical stability after storage at room temperature with exposure to light [4].

Clonidine is an imidazoline-derivative used as hypotensive agent, since it is a centrally-acting α2-adrenergic agonist; it is also an analgesic (when administered epidurally, it produces a dose-dependent analgesia), sedative, and anxiolytic drug, properties that allow its use in anesthesia and intensive care. Clonidine appeared to be sensitive to acidic and oxidative conditions but remained stable under dry heat 60 °C and basic hydrolysis conditions [5].

Sufentanil is a synthetic opioid with a morphinomimetic structure. It is a very potent analgesic, used in intensive care units for its sedative properties. Sufentanil seems to be susceptible to many stress conditions. A. Jappinen et al. [6] observed how the increase in temperature affects the stability of Sufentanil: at 4 °C, a Sufentanil citrate solution maintained chemical stability for 23 days, but at 21 °C, it was stable for 3 days. In addition, Sufentanil was fragile under acidic and basic hydrolysis, oxidative, or light stress conditions [6]. 

Loxapine, a dibenzoxazepine derivative, is a conventional antipsychotic (dopaminergic antagonist), mainly used in the treatment of schizophrenia and associated anxiety, agitation, and irritability. A very limited study on the drug stability was found in the literature.

An extensive study of the literature revealed no data regarding the stability behaviour of at least three of the selected drugs mixed. 

This research is a comprehensive physicochemical compatibility study of intensive care units selected drugs (Sufentanil, Clonidine, Loxapine, Midazolam, and Ketamine), with the aim of understanding if it is possible to encompass all the combinations of the five molecules in the same injection solution, either entirely or partially, in order to reduce the number of syringes requested. From a medical staff perspective, drugs’ combination stability must be of at least two days to cover the medication administration period in intensive care units.

## 2. Materials and Methods

### 2.1. Materials

#### 2.1.1. Chemicals and Reagents

Midazolam (5 mg mL^−1^) and sufentanil (5 µg mL^−1^) were provided by Mylan^®^ Laboratory (Saint-Priest, France), clonidine (Catapressan^®^ 0.15 mg mL^−1^) was obtained from Boehringer Ingelheim (Paris, France), and loxapine (Loxapac^®^ 25 mg mL^−1^) and ketamine (50 mg mL^−1^) were supplied by Eisai^®^ (Courbevoie, France) and Panpharma^®^ (Luitré-Dompierre, France), respectively. ChemAxon prediction and calculation software (MarvinSketch version 15.11.9, Cambridge, MA, USA) was used to predict the drugs’ main physicochemical properties such as pK_a_ and logP (Table 1). Reference standards of the five products were purchased from Sigma-Aldrich (St Quentin-Fallavier, France). Formic acid 99–100% (AnalaR Normapur^®^) was supplied by VWR International (Fontenay-sous-Bois, France). HCl 1 M and NaOH 10 M were obtained by VWR International. Analytical grade methanol came from Sigma–Aldrich (St Quentin-Fallavier, France). Sterile water (Versylene^®^), sodium chloride (0.9% NaCl), and glucose 5% (G5%) were obtained from Fresenius Kabi France SA (Sevres, France). Polypropylene syringes were purchased from Becton Dickinson^®^ (Rungis, France). Glass vials used for stability study were provided by Interchim^®^ (Montluçon, France).

#### 2.1.2. Instrumentation

A reversed-phase liquid chromatography (LC) method was implemented to study drug combinations in the presence of potential degradation products. The LC system (Dionex, Les Ulis, France) consists of a quaternary pump, a vacuum degasser, a photo-diode array (PDA) detector, and an autosampler, piloted by Chromeleon^®^ software version 6.80 SR11 (Dionex, Les Ulis, France). The pH of each drug product and its mixtures was measured using a Mettler Toledo SevenCompact™ pH meter (Columbus, OH, USA).

### 2.2. Methodology

#### 2.2.1. Stress Testing and High Performance LC Method Optimization and Validation 

##### Degradation Protocol 

Prior to the method validation, stress testing was performed to develop the method, according to International Council on Harmonisation (ICH) guidelines for a better understanding of the drugs’ intrinsic stability. Each drug product and mixture of 5 compounds were subjected to high temperature (60 ± 0.5 °C), acidic and basic hydrolysis (HCl 1N and NaOH 1N, respectively, stored at 22 ± 3 °C) and oxidative (0.5% H_2_O_2_ stored at 22 ± 3 °C) stress conditions. A dilution factor of 10 was applied to each drug product. Samples were analyzed at days 0, 1, 2, 5, and 7 and the chromatograms were compared with the ones of control samples. For the purpose of interpretation, a 20% degradation level was considered. 

##### Method Optimization 

The aim of the optimization was to develop a single method able to separate the 5 drugs with appropriate resolution factor and without interferences with potential degradation products. In order to achieve this goal, several chromatographic parameters such as mobile phase composition, column, pH, temperature or detection wavelength were appropriately chosen and optimized. Separation was considered as appropriate if resolutions between two peaks were all superior to 1.5. A desirable separation and resolution was obtained using a mobile phase composed of methanol (solvent A) and 0.1% formic acid in water (solvent B) set in a gradient mode (0–10 min: 80% A; 10–17 min: 80% → 30% A; 17–22 min: 30% → 80% A); the flow rate was set at 0.8 mL/min and the injection volume at 40 μL. The selected column was an Agilent Zorbax SB-C8 column, 5µm × 4.6 × 250 mm, maintained at 25 °C ± 1 °C. The vials were thermostated at 8 °C during the LC process. Due to the shortening of width of Sufentanil absorption in the UV region, the detection wavelength was set at 205 nm. The other 4 molecules were detected and quantified at 205 and 225 nm. 

##### Method Validation

(a) Validation protocol

Relevant validation parameters (specificity, linearity, accuracy, precision, and limits of detection and quantification) as per ICH Q2R1 [7] and SFSTP guidelines [8] were evaluated.

(a.1) Specificity

Specificity was established by observing the separation of the 5 main peaks eventually with degradation products. Peak purity was analyzed using a PDA detector.

(a.2) Linearity and Accuracy

Linearity and accuracy were determined over three days by three operators analyzing different concentrations of each drug. Seven-point calibration curves (70–80–90–100–110–120–130%) around the target concentration were considered. For each drug, linear adjustment was validated by means of Fischer’s statistical analysis and ANOVA variance analysis. An appropriate quantification method for each drug was decided with the support of a Student’s *t*-test comparison of the linear regression slopes, between the drug solely and in presence of the other ones. 

(a.3) Precision

For each drug, intraday and interday repeatability were assessed for a target concentration by assaying six samples solutions on three consecutive days.

(a.4) LOD and LOQ

Limits of detection (LOD) and quantification (LOQ) were established by the graphical method considering the signal–background noise ratio of 3:1 and 10:1, respectively.

(b) Stability study

The finding of an optimized protocol allowed the execution of a stability study. Applying the number of combinations formula (Equation (1)), 26 possible combinations, named from M1 to M26, were obtained: 10 for mixture of 2 or 3 drugs, 5 for 4 drugs, and 1 for combination of all the 5 drugs (Table 2).

(1) $$C_{\left(n,k\right)}=\left(\begin{array}{c} n\\ k \end{array}\right)=\frac{n!}{k!\left(n-k\right)!}\;$$

where *k* is the number of combinations and *n* the number of elements (drugs).

For each combination, samples were made up in triplicate and allocated in 4 mL hermetically sealed glass vials or polypropylene syringe. The study was performed considering two steps. First, a compatibility study was realized employing the five pure drugs products. Their final concentrations in each combination was related to the number of drugs contained within it, so a dilution factor of 1/2, 1/3, 1/4 and 1/5 was applied respectively for the combinations of 2, 3, 4, and 5 drugs (Table 3). The second step considered the execution of a stability study in real operating conditions; therefore, NaCl 0.9% and Glucose 5% were used as diluting solvents to recreate the real set-up of administration (Table 3). The samples were prepared in glass vials and polypropylene syringe, then stored at 25 °C.

Different parameters were observed to verify the stability of the preparations:

(b.1) Content variation

For the first step described above, high performance LC was performed to analyze the content of each combination at days 0, 2, 3, 7, and 14, while for the second one, the study was conducted until day 7, in order to observe any variation of the concentrations out of the range of ± 5%. For each mixture, the working solution was prepared by diluting stock solution in water in sort to obtain a final concentration of 1 μg mL^−1^, 5 μg mL^−1^, 5 μg mL^−1^, 25 μg mL^−1^, and 30 μg mL^−1^, respectively, for sufentanil, ketamine, midazolam, loxapine, and clonidine. These results are validated by monitoring the appearance of degradation products following the ICH Q3B [9] recommendations.

(b.2) pH and visual control

The pH of each drug product taken individually and the 26 combinations of pure products (step 1) was measured to find out any change of the parameter, since a stable value is an indicator of chemico-physical stability. pH was measured at room temperature at days 0, 3, 7 and 14. Samples were inspected against a light and dark background to monitor clearness and turbidity.

(b.3) Statistical interpretation

All statistical tests were performed using GraphPad Prism V 9.3.1.471 for Windows (GraphPad Software, La Jolla, CA, USA) using a two-way ANOVA.

## 3. Results

### 3.1. Method Validation 

#### 3.1.1. Specificity 

**Figure 1:**
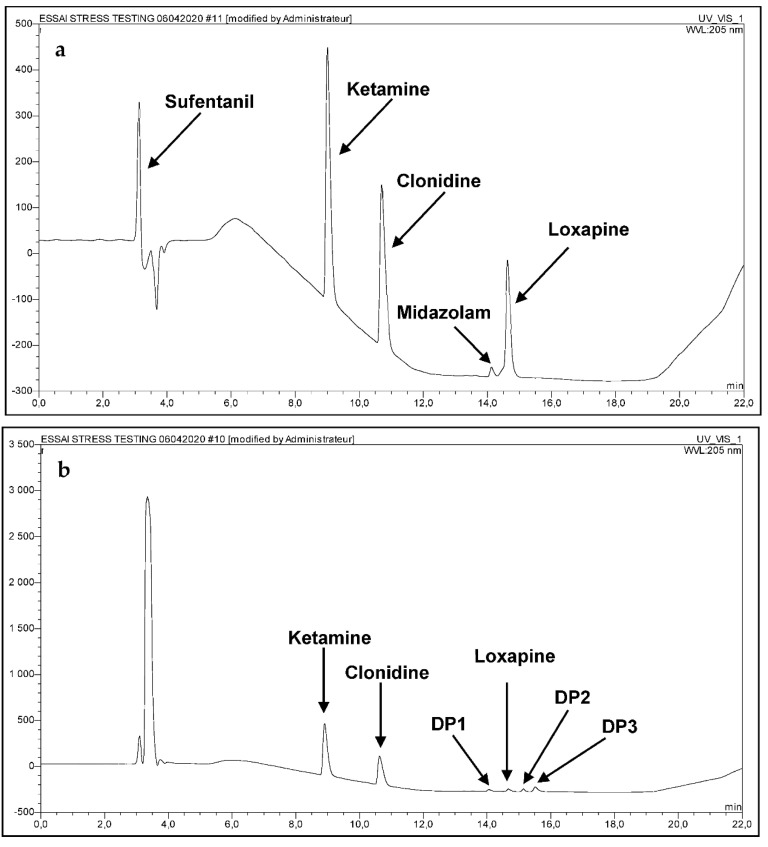

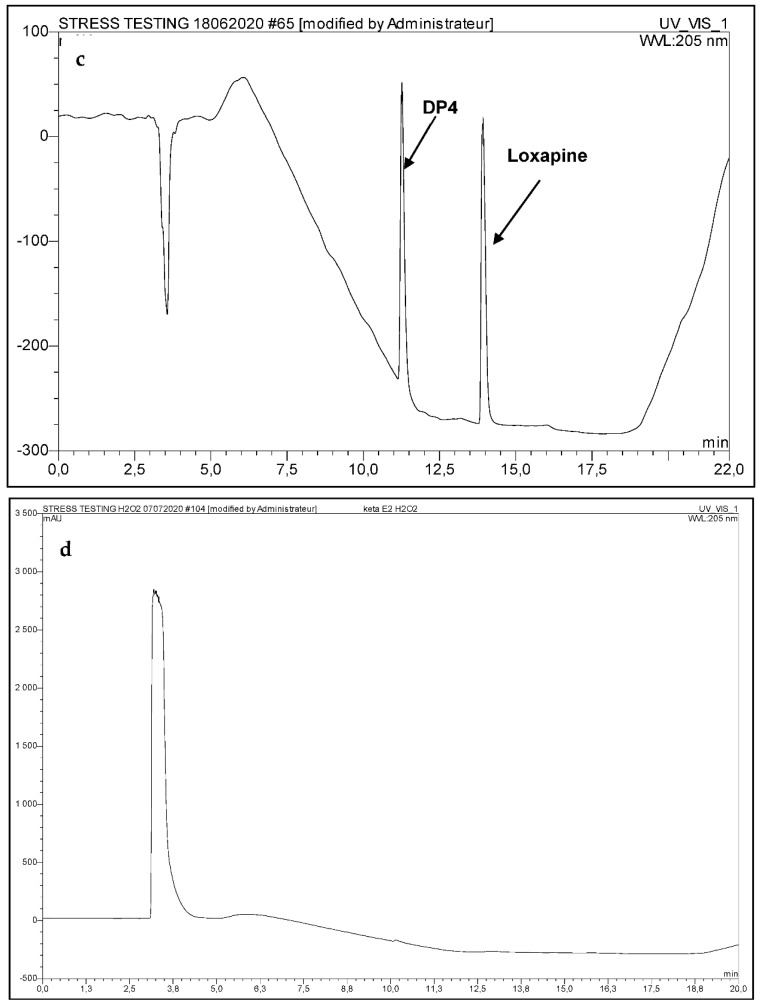
(**a**) Chromatogram of the five drugs in water (**b**) chromatogram of the five drugs under oxidative conditions (**c**) chromatogram of Loxapine under acidic and thermal conditions (60 °C) after 24 h exposure (**d**) Oxidative media blank sample.

The complexity of method validation in this study was to develop a single method capable of identifying and quantifying simultaneously the five molecules in the presence of the degradation products. The developed analytical procedure reached the ability to separate not only the five molecules, but also their degradation products generated under stress conditions. Hence, the absence of interference validated the stability, indicating power and the suitability of the method to identify and quantify all the molecules in question (Figure 1). As Sufentanil, the most hydrophilic of the five products, is eluted after only 3 min of running, (Figure 1), a complementary method, as described by Lambropoulos et al. [10], was developed for its content determination within the mixtures.

#### 3.1.2. Linearity, Accuracy, and Precision

Validation results are summarized in the tables below (Table 4). The ANOVA non-parametric statistical test was used to verify homogeneity of variance for linearity, accuracy, and precision. 

For all the calibration curves, a good linear relationship was found between the signal and the concentration (F_calc_ > 4.38 = F_critical_, 5%; (1;19)) and the Fischer’s statistical analysis demonstrated the absence of deviation from linearity (F_calc_ < F_critical_, 5%; (5, 14)). 

Aside from the curve of Clonidine, the intercept value of the other drugs was statistically different from zero (t_calc_ > 2.093 = t_critical_ 5%, 19 degrees of freedom), making it difficult to use a single-point calibration for routine analysis. Methods were found to be precise and accurate (Table 4 and Supplementary Materials Figure S1A–E).

For each drug, the Student’s *t*-test based on the standard error of regression was used to compare the slopes and the y-intercepts of the regression lines of the drug solely, and in the presence of the other drugs. A matrix effect associated with the presence of the other drugs was highlighted with Ketamine and Midazolam (t_calc_ > 2.042 = t_critical_ 5%, 38 degrees of freedom). As a result, a single calibration curve incorporating the five molecules was performed for the stability study.

### 3.2. Impact of Stress Conditions on the Drugs’ Stability

For better understanding of the physicochemical behavior of the drugs in the mixture, upstream stress testing provided a major contribution in terms of extrapolating and/or mitigating the impact of such stress parameters on the mixture stability.

#### 3.2.1. Physical Stability

Under alkaline conditions, Loxapine, Midazolam, and Ketamine generated an immediate precipitation, which in the case of Ketamine gave rise to the formation of crystals since the first day of exposure. The acidification visibly affected only Loxapine, causing a precipitation.

#### 3.2.2. Chemical Degradation

##### Acidic and Basic Hydrolysis

Approximately 90% of the initial Ketamine concentration was lost after 24 h under alkaline conditions, due to precipitation and crystallization, but no degradation product was observed. However, the acidic catalysis had no impact on the Ketamine concentration over the study period (Supplementary Materials Figure S2B).

Despite the precipitation observed with the Loxapine drug product under acidic condition, the content of the API was barely degraded, generating a degradation product at room temperature (25 °C) after 24 h exposure. The degradation was potentiated at 60 °C, ending up by completely converting Loxapine to its degradation product after only five days of exposure (Supplementary Materials Figure S2C). 

Under the basic condition, the concentration of Midazolam decreased down to 50% after only 24 h of exposure. The acidic stress had a low impact on the drug (85% of the concentration was found), generating a degradation product less lipophilic than Midazolam (RT at 10.1 min) (Supplementary Materials Figure S2D). 

Neither acidic nor basic catalysis generated loss of Clonidine and Sufentanil, where concentrations remained greater than 97% of their initial concentrations after seven days of exposure (Supplementary Materials Figure S2A–E).

##### Thermal Degradation

Thermal stress did not generate significant loss in any of the molecules over the seven-day study period (Supplementary Materials Figure S2A–E).

##### Oxidative Degradation

**Figure 2:**
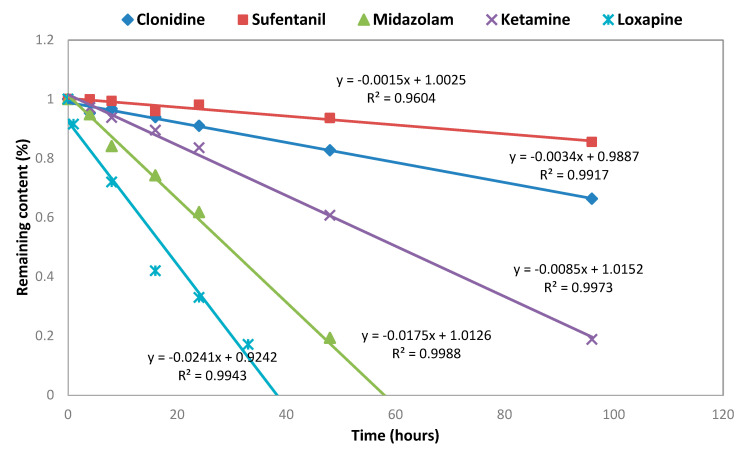
Degradation kinetic under oxidative conditions.

The five molecules reacted differently to the oxidative stress. Clonidine and Sufentanil showed less sensitivity, as the half-life (t_1/2_) was 138 and 468 h, respectively. Ketamine was more degraded, as the concentration decreased down to 50%, after 64 h exposure (kinetics constant (k) of 1.5 × 10^−3^ µmol L^−1^ h^−1^). Loxapine and Midazolam was revealed to be extremely fragile towards oxidative conditions, giving a half-life (t1/2) of 17 and 28 h, respectively, and a kinetics constant (k) of 2.2 × 10^−3^ and 2.8 × 10^−4^ µmol L^−1^ h^−1^, respectively. Loxapine generated three more lipophilic degradation products. All the degradations followed zero-order kinetics models (Figure 2).

### 3.3. Compatibility Study 

#### 3.3.1. Content Variation

**Figure 3:**
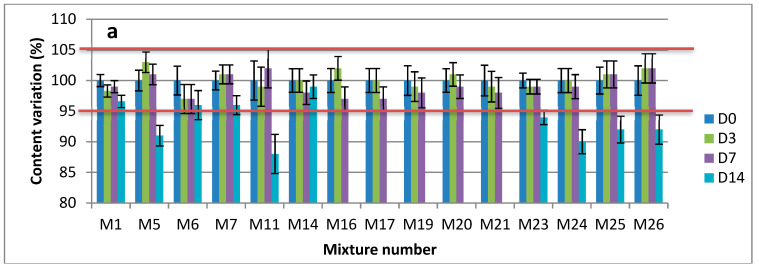

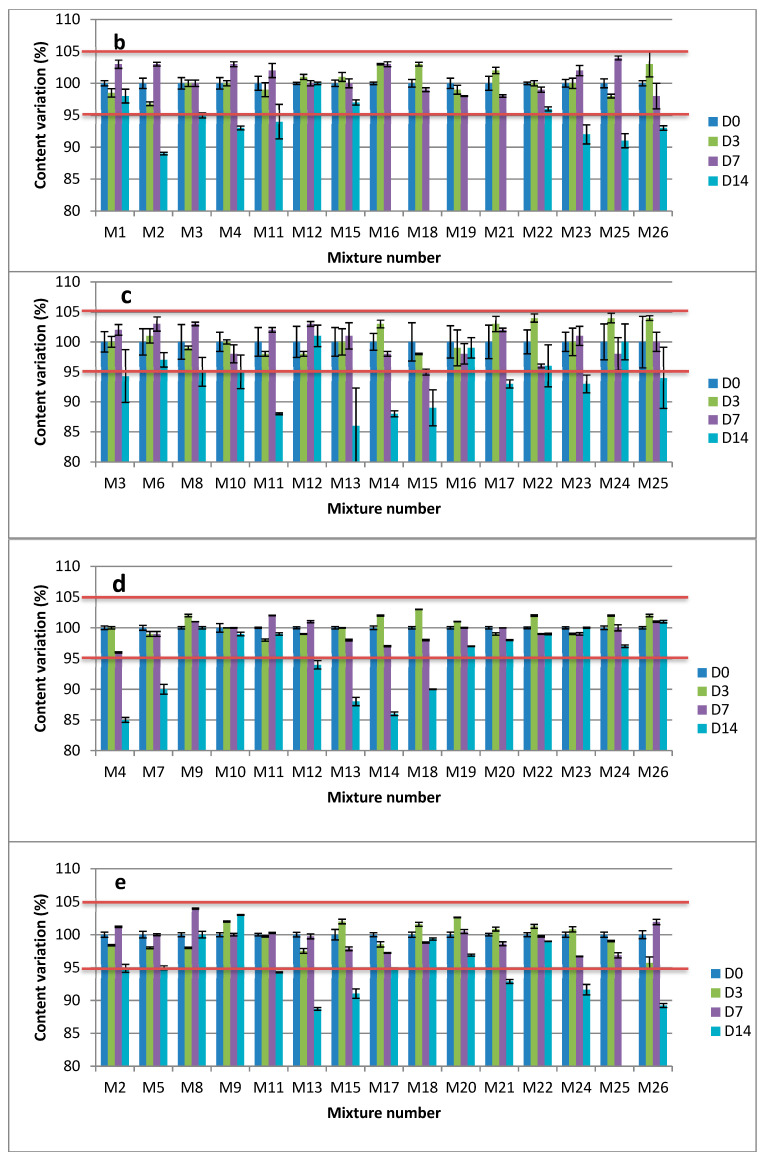
Observed concentrations of pure drugs (as mean percentage of initial concentration ± SD) in mixtures at 22 ± 3 °C: (**a**) Clonidine; (**b**) Sufentanil; (**c**) Loxapine; (**d**) Midazolam; (**e**) Ketamine.

The initial concentration of each drug was indicated as 100% and all the following concentrations were expressed as a percentage of the initial concentration. With the acceptance criteria of ±5% from the initial concentration, all of the 26 possible combinations of the five drugs displayed adequate stability at 25 °C: at least 3 days and 7 days, respectively, for the dilution in 0.9% NaCl or G5%, and the pure drug products’ mixtures (Figure 3 and Supplementary Materials Figure S3A,B). Furthermore, no degradation product was observed over the study period. The same applies for the containers, where no interaction with polypropylene or glass vials was established.

#### 3.3.2. pH and Visual Control

All the solutions remained clear, colorless, and showed no visible particles during the whole study. The variation of the pH of all the mixtures between D0 and D14 was lower than 0.4 pH unit.

## 4. Discussion

No previous LC method separating and quantifying simultaneously the five molecules in the presence of their degradation products has been described. Despite the differences in the chemical and physical properties and concentration levels between the analytes, the gradient mode provided satisfactory results in terms of linearity, accuracy (SD < 3%), and precision (interday and intraday variation < 3%), demonstrating the stability indicating capability of the method.

All the 26 combinations prepared in glass vials or polypropylene syringes, whether as a result of the mixture of pure drugs solutions or diluted in a 0.9% NaCl or G5%, maintained at least 95% of the initial concentration for each molecule for 7 and 3 days, respectively, at 25 °C. When gathering all the results of assays (expressed in recovery, %) obtained at day 7 as a function of drug substance and the vehicle (NaCl 0.9% or G5%), two-way ANOVA showed that both the drug substance (*p* < 0.0001) and the choice of vehicle (*p* = 0.0179) had effects on the assay value and interaction was found to be significant (*p* < 0.0001). The lowest assay value (mean = 98.7%; SD = 1.54%) was obtained when the considered drug and vehicle were Midazolam and G5%. 

Overall, apart from Loxapine for which little information was available, all the molecules were described to be compatible with polypropylene materials [2,11,12]. However, as described by Roos et al. [11], the interaction between Sufentanil and the plasticizer (DEHP) of polyvinyl chloride must be avoided. 

Regarding pH, since the drug products present a similar pH, between 4 and 6, their mixtures do not show a considerable variation in pH value to one of single molecules. In view of pK_a_, in the five molecules (Table 1), at pH 6, calculations form ChemAxon showed that 90% to 99% of species are almost exclusively in the ionized form (Supplementary Materials Figure S4), thus substantially reducing the risk of interaction with the containers. 

The strong acidity of the Midazolam drug product (pH at 3.7) increases its stability as it prevents the diazepine ring from opening, which protects the solution against photo-decomposition [13]. However, the significant increase in pH of its mixtures (pH values between 4 and 6) is not accompanied by any degradation of Midazolam. The stability indicating method prevented analytical problems of content determination of Midazolam related to pH variation (Supplementary Materials Figure S4).

In basic conditions, phenomena of precipitation/crystallization were observed with Loxapine, Midazolam, and Ketamine, which indicate the need to avoid mixtures with basic molecules or other drugs not evaluated in the study or similar conditions. Furthermore, under alkaline conditions, most of the drug products were in their uncharged forms, which can magnify the risk of interactions. 

After three days of exposure, Loxapine in 0.9% NaCl or G5% exhibited a shorter stability period, along with a content variation of ±15% at day 7. The absence of degradation products associated with content fluctuation seems to indicate that Loxapine presents a low physical stability after D3, probably due to its lipophilic properties. Conversely, Ketamine, Clonidine, Sufentanil, and Midazolam remained stable in the same conditions until day 7. 

All our experiments were conducted without any protection from daylight. However, in view of the sensitivity of the five molecules to oxidation, it is thus advocated to protect the mixtures from light, so as to avoid indirect photolysis in the presence of oxygen [14]. 

Although the initial design of the study target of almost two-day mixture stability was achieved, the clinical use of such drug combinations requires upstream preparation under validated aseptic conditions, within hospital pharmacy sterile suites.

## 5. Conclusions

This study provided extensive information regarding the compatibility and stability behavior of Midazolam, Loxapine, Clonidine, Sufentanil, and Ketamine drugs products. The single high performance LC method developed and optimized demonstrated its stability-indicating capability to analyze accurately the five molecules in the presence of their degradation products without interferences. All the 26 possible combinations of the five drugs displayed satisfactory stability at 25 °C: at least 3 days and 7 days, respectively, for dilution in 0.9% NaCl or G5%, and pure drug products’ mixtures. In view of the increased consumption of pharmaceuticals related to the COVID pandemic, a combination of more than two drugs offers the advantage of minimizing the individual doses and reducing unwanted side-effects [15,16]. Hence, it opens up the possibility of combining the give drugs in one single syringe, especially under the current circumstances associated with an important shortage of programmable syringe pumps, syringes, and pharmaceuticals. The finding that most of the five molecules underwent physicochemical degradation under oxidation and/or alkaline is important to understand the optimal operative conditions that guarantee the quality of the preparations. This data will also be useful for the management of critically ill patients.

## Figures and Tables

**Table 1:** Summary of the drugs products and active pharmaceutical ingredient (API) properties.

Drug Product	Route of Administration	Concentration	Excipients	Laboratory	API Structure	logP	pK_a1_	pK_a2_
Ketamine Chlorhydrate	Intramuscular or intravenous	50 mg mL^−1^	Chlorobutanol, water for injection	Panpharma	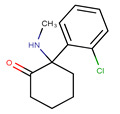 Ketamine	2.65	7.16	-
Midazolam Chlorhydrate	Intramuscular, intravenous or rectal	5 mg mL^−1^	NaOH, NaCl, HCl, water for injection	Mylan	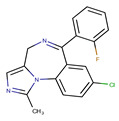 Midazolam	3.33	3.48	6.57
Clonidine chlorhydrate (Catapressan^®^)	Intramuscularor intravenous	0.15 mg mL^−1^	NaCl, HCl, water for injection	Boehring	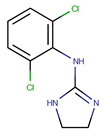 Clonidine	2.49	8.16	-
Sufentanil Citrate	Intravenous and epidural	5 μg mL^−1^	NaOH, NaCl, HCl, water for injection	Mylan	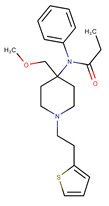 Sufentanil	3.61	8.86	-
Loxapine (Loxapac^®^)	Intramuscular	25 mg mL^−1^	Polysorbate 80, Propylene glycol, HCl, water for injection	EISAI	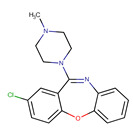 Loxapine	3.46	1.02	7.18

**Table 2:** Drugs mixtures qualitative composition.

Mixture n°	Composition	Mixture n°	Composition
M1	Sufentanil + Clonidine	M14	Loxapine + Clonidine + Midazolam
M2	Sufentanil + Ketamine	M15	Loxapine + Sufentanil + Ketamine
M3	Sufentanil + Loxapine	M16	Loxapine + Sufentanil + Clonidine
M4	Sufentanil + Midazolam	M17	Loxapine + Clonidine + Ketamine
M5	Clonidine + Ketamine	M18	Midazolam + Sufentanil + Ketamine
M6	Clonidine + Loxapine	M19	Midazolam + Sufentanil + Clonidine
M7	Clonidine + Midazolam	M20	Midazolam + Clonidine + Ketamine
M8	Ketamine + Loxapine	M21	Sufentanil + Ketamine + Clonidine
M9	Ketamine + Midazolam	M22	Loxapine + Midazolam + Sufentanil + Ketamine
M10	Loxapine + Midazolam	M23	Loxapine + Midazolam + Sufentanil + Clonidine
M11	Midazolam + Loxapine + Clonidine + Ketamine + Sufentanil	M24	Loxapine + Midazolam + Ketamine + Clonidine
M12	Loxapine + Midazolam + Sufentanil	M25	Loxapine + Sufentanil + Ketamine + Clonidine
M13	Loxapine + Ketamine + Midazolam	M26	Midazolam + Sufentanil + Ketamine + Clonidine

**Table 3:** Drugs mixtures quantitative composition.

Drug	Step 1: Drug Products Compatibility and Stability Study					Step 2: Stability Study in Clinical Use
	Initial Cc (µg mL^−1^)	Final Cc 2 Drugs Comb (µg mL^−1^)	Final Cc 3 Drugs Comb (µg mL^−1^)	Final Cc 4 Drugs Comb (µg mL^−1^)	Final Cc 5 Drugs Comb (µg mL^−1^)	Concentration after Dilution with NaCl 0.9% or G5% (µg mL^−1^)
Ketamine	50,000	25,000	16,670	12,500	10,000	500
Clonidine	150	75	50	37.5	30	5
Loxapine	25,000	12,500	8330	6250	5000	500
Midazolam	5000	2500	1670	1250	1000	500
Sufentanil	5	2.5	1.67	1.25	1	1

Cc = Concentration; Comb = Combination.

**Table 4:** LC method validation parameters.

Paramethers	Ketamine	Ketamine *	Midazolam	Midazolam *	Loxapine	Loxapine *	Clonidine	Clonidine *	Sufentanil	Sufentanil *
Linearity										
Concentration range (µg mL^−1^)	1 to 10	1 to 10	5 to 50	5 to 50	5 to 50	5 to 50	21 to 39	21 to 39	0.7 to 1.3	0.7 to 1.3
Slope	2.78	2.52	3.82	4.26	4.21	4.20	6.16	6.21	14.44	14.41
SD Slope	0.04	0.042	0.03	0.01	0.09	0.09	0,08	0.07	0.68	0.45
y-intercept	−1.41	−0.87	−14.15	−8.66	−20.01	−22.23	1.40	3.80	19.67	23.04
SD y-intercept	0.22	0.25	0.77	0.23	2.88	2.68	2.36	2.28	0.69	0.46
Correlation coefficient (R)	0.99	0.99	0.99	0.99	0.99	0.99	0.99	0.99	0.96	0.98
LOD (µg mL^−1^)	0.30	0.30	1.0	1.0	1.0	1.0	0.20	0.20	0.002	0.002
LOQ (µg mL^−1^)	0.90	0.90	3.0	3.0	3.0	3.0	0.60	0.60	0.006	0.006
Accuracy										
%Recovery	99.14	101.26	99.60	99.75	97.64	100.67	100.07	99.93	99.97	99.96
SD	4.62	3.88	2.74	1.34	2.49	3.23	1.20	1.01	4.34	3.09
Precision										
Repeatability	3.17%	1.46%	1.68%	0.73%	1.31%	2.40%	1.55%	2.36%	2.33%	2.68%
Reproducibility	2.89%	1.33%	1.54%	0.67%	1.50%	3.10%	1.42%	2.15%	2.13%	2.45%

* in presence of the other molecules.

## Data Availability

The study did not report any data.

## Supplementary Materials

The following supporting information can be downloaded at: https://www.mdpi.com/article/10.3390/pharmaceutics14030550/s1, Figure S1: Method validation (A) Clonidine; (B) Ketamine; (C) Loxapine; (D) Midazolam; (E) Sufentanil; Figure S2: Observed content variation under stress conditions (as mean percentage of initial concentration±SD) for each drug: (A) Clonidine; (B) Ketamine; (C) Loxapine; (D) Midazolam; (E) Sufentanil; Figure S3: (A) Drugs mixture diluted in NaCl: (a) Clonidine; (b) Sufentanil; (c) Loxapine; (d) Midazolam; (e) Ketamine, (B) Drugs mixture diluted in G5%: (a) Sufentanil; (b) Clonidine; (c) Midazolam; (d) Ketamine; (e) Loxapine; Figure S4: Microspecies distribution of the 5 drugs individually according to the pH.
