# Supplementary material for: Therapeutic Treatment Plan Optimization during the COVID-19 Pandemic: A Comprehensive Physicochemical Compatibility Study of Intensive Care Units Selected Drugs
Source: Pharmaceutics. 2022 Feb 28;14(3):550. doi: 10.3390/pharmaceutics14030550 (PMC8952813; doi:10.3390/pharmaceutics14030550)
Supplement: Supplementary file 1 [file pharmaceutics-14-00550-s001.zip › Supplementary materials Figure S1A-Clonidine method Validation.pdf]

Linearity

CLONIDINE drug product

Essay	Observed AUC (mAu)	Concentration (µg/L)	Variance s²	Estimated AUC	Adjusted AUC	Confidence interval
70% D1	128,4622	21	5,366300103	130,7374619	128,4622	-1,071906914
70% D2	127,2393	21		130,7374619	127,2393	-1,648031782
70% D3	131,7208	21		130,7374619	131,7208	0,463263988
80% D1	149,0511	24	1,21167028	149,2143	149,0511	-0,076885746
80% D2	149,7241	24		149,2143	149,7241	0,240173733
80% D3	151,2029	24		149,2143	151,2029	0,936856581
90% D1	170,1337	27	3,82240092	167,6911381	170,1337	1,150724225
90% D2	166,4311	27		167,6911381	166,4311	-0,593621131
90% D3	167,1937	27		167,6911381	167,1937	-0,234349871
100% D1	188,9042	30	0,37068403	186,1679762	188,9042	1,289072354
100% D2	187,797	30		186,1679762	187,797	0,767455334
100% D3	188,7895	30		186,1679762	188,7895	1,235035619
110% D1	202,9627	33	7,455189213	204,6448143	202,9627	-0,792466981
110% D2	201,0545	33		204,6448143	201,0545	-1,691446025
110% D3	206,4397	33		204,6448143	206,4397	0,845595139
120% D1	222,5938	36	2,185793543	223,1216524	222,5938	-0,248678456
120% D2	222,1724	36		223,1216524	222,1724	-0,447205743
120% D3	224,9177	36		223,1216524	224,9177	0,846142528
130% D1	240,1313	39	6,705049103	241,5984905	240,1313	-0,691213443
130% D2	238,8034	39		241,5984905	238,8034	-1,316805243
130% D3	243,8024	39		241,5984905	243,8024	1,038291834

Linear regression (LINEST function)

Slope b	6,158946032	1,399595238	Intercept a
S slope	0,077199311	2,361844766	S Intercept
R²	0,997023724	2,122630124	S res
F	6364,817361	19	DOF
Regression sum of squares	28677,05786	85,6056142	Residual sum of squares

CHECKING THE FIT TO THE LINEAR MODEL n=3 k=7

Cochran's C test: Study of the homogeneity of a series of variances

k : number of samples per test

C test = max s²/sum s²

C test	**0,274925886**		0,274925886
Cochran's critical C, α = 5%	0,561		
Maximum S²	7,455189213		
Sum s²	27,11708719		

The variances are homogeneous at the risk of 0.05% because 0.275<0.561

Calculation of experimental variance s²exp

s²exp = 3,873869599

Calculation of misfit variance

SCE aj = 46,86691821

s² aj = 15,62230607

Fisher Test

H0: Not significantly different

H1: Significantly different

Fexp 4,032739272 **F threshold limit 5% (5, 14)** 2,958248913 **p-value** 0,017783205

Conclusion p-value > 5%, therefore no rejection of H0: Fit to the validated linear model.

DEPENDENCE VERIFICATION. F(1, n - 2)

H0: The variances are not significantly different

H1: The variances are significantly different

F exp **6364,817361** **F threshold 5% (1, 19)** 4,38 **p-value** 1,80856E-25

Conclusion Dependence is validated as H0 is rejeted, p-value<5%

VALIDATION OF THE PROPORTIONAL MODEL

Regression of CLONIDINE within the mixture: Comparison of the y-intercept to 0

IaI	1,399595238
Standard deviation of the inte	2,361844766

Student Test

**H0:**	The y-intercept is equal to 0
**H1:**	The y-intercept is different from 0
**t exp**	**0,592585617**
**Nb of DOF (N-2)**	19
**t threshold (5%, student)**	2,093

Conclusion texp < t threshold→ No rejection of H0 at 5% risk. The y-intercept is not significantly different from 0

the correlation line passes through the origin

CLONIDINE Pure drug Product Regression: Comparing the Intercept to 0

IaI	3,803790476
Standard deviation	2,2821159

Student Test

**H0:**	The y-intercept is equal to 0
**H1:**	The y-intercept is different from 0
**t exp**	**1,666782338**
**Nb of DOF (N-2)**	19
**t threshold (5%, student)**	2,093

Conclusion texp < t threshold → No rejection of H0 at 5% risk. The y-intercept is not significantly different from 0

the correlation line passes through the origin

CALCULATED LIMIT OF DETECTIO	**3,446417801**
CALCULATED LIMIT OF QUANTIFICATI	**1,137317874**

COMPARISON OF CLONIDINE IN THE MIXTURE AND THE PURE DRUG PRODUCT REGRESSIONS

Comparison of Student slopes

**H0:**	No difference between the two slopes
**H1:**	Difference between the two slopes
**texp**	**0,505706633**
**Nb of DOF (N1+N2-4)**	38
**t threshold (5%, student à 38**	2,042

Conclusion texp < t threshold → No rejection of H0 at 5% risk, no significant difference between the 2 slopes.

COMPARISON OF STUDENT INTERCEPTS

**H0:**	No difference between the two intercepts
**H1:**	Difference between the two intercepts
**t exp**	**0,732035602**
**Nb of DOF (N1+N2-4)**	38
**t threshold (5%, student à 38 c**	2,042

Conclusion texp < t threshold→ No rejection of H0 at 5% risk therefore no systematic error.

SIGNIFICANCE TEST OF CLONIDINE IN THE MIXTURE FORM CORRELATION COEFFICIENT (Pearson correlation coefficient test)

R reconstituted form: 0,998510753

**H0:**	R=0
**H1:**	R different from 0
**texp**	**79,77980547**
**Nb of DOF**	19
**t threshold (5%, student)**	2,093

Conclusion texp > t threshold→ Rejection of H0 at 5% risk, existence of a significant linear link between the variables

SIGNIFICANCE TEST OF CLONIDINE PURE PRODUCT CORRELATION COEFFICIENT (Pearson correlation coefficient test)

R pure product 0,99863354

**H0 :**	R=0
**H1 :**	R different from 0
**texp**	**83,29480025**
**Nb of DOF:**	19
**t threshold (5%, student)**	2,093

Conclusion texp > t threshold → Rejection of H0 at 5% risk, existence of a significant linear link between the variables

Linearity

CLONIDINE within the mixture

Essay	Observed AUC (mAu)	Concentration (µg/L)	Variance s²	Estimated AUC	Adjusted AUC	Confidence interval
70% D1	134,9275	21	0,46850889	134,2816905	134,9275	0,314879056
70% D2	134,3452	21		134,2816905	134,3452	0,030965506
70% D3	133,5634	21		134,2816905	133,5634	-0,350218786
80% D1	151,8987	24	1,997169603	152,9213905	151,8987	-0,498635898
80% D2	152,5736	24		152,9213905	152,5736	-0,169573122
80% D3	154,6131	24		152,9213905	154,6131	0,824831283
90% D1	168,6953	27	6,78426988	171,5610905	168,6953	-1,397281036
90% D2	173,8201	27		171,5610905	173,8201	1,101431244
90% D3	170,4483	27		171,5610905	170,4483	-0,542566193
100% D1	190,2357	30	4,216844103	190,2007905	171,596	0,017020929
100% D2	191,4522	30		190,2007905	191,4522	0,610153049
100% D3	187,4468	30		190,2007905	187,4468	-1,342770415
110% D1	206,9355	33	5,324505703	208,8404905	206,9355	-0,928821241
110% D2	211,0744	33		208,8404905	211,0744	1,08919317
110% D3	210,7729	33		208,8404905	210,7729	0,942190018
120% D1	229,7488	36	0,273347583	227,4801905	229,7488	1,106111941
120% D2	228,7038	36		227,4801905	228,7038	0,596598529
120% D3	229,1943	36		227,4801905	229,1943	0,835752911
130% D1	244,5394	39	11,14275396	246,1198905	244,5394	-0,770603918
130% D2	247,9516	39		246,1198905	247,9516	0,893091453
130% D3	241,276	39		246,1198905	241,276	-2,361748481

Linear regression (LINEST function)

Slope b	6,213233333	3,803790476	Intercept a
S slope	0,074593292	2,2821159	S Intercept
R²	0,997268947	2,050976434	S res
F	6938,023749	19	DOF
Regression sum of squares	29184,82695	79,9235823	Residual sum of squares

CHECKING THE FIT TO THE LINEAR MODEL n=3 k=7

Cochran's C test: Study of the homogeneity of a series of variances

k : number of samples per test

C test = max s²/sum s²

C test	**0,36887498**		0,36887498
Cochran's critical C, α = 5%	0,561		
Maximum S²	11,14275396		
Sum s²	30,20739972		

The variances are homogeneous at the risk of 0.05% because 0,369<0,561

Calculation of experimental variance s²exp

s²exp = 4,315342818

Calculation of misfit variance

SCE aj = 36,77015412

s² aj = 12,25671804

Fisher Test

H0: Not significantly different

H1: Significantly different

Fexp 2,840265202 **F threshold limit 5% (5, 14)** 2,958248913 **p-value** 0,05647278

Conclusion p-value > 5%, therefore no rejection of H0: Fit to the validated linear model

DEPENDENCE VERIFICATION. F(1, n - 2)

H0: The variances are not significantly different

H1: The variances are significantly different

F exp **6938,023749** **F threshold limit 5% (1, 19)** 4,38 **p-value** 7,9896E-26

Conclusion Dependence is validated as H0 is rejeted, p-value<5%

Precision

CLONIDINE drug product

Essay Number	AUC (mAu*min)	Concentration (µg/mL)	Observed concentration (µg/mL)	Recovery Rate	
Day 1-1	178,9556	30	28,82895935	1,039034688	Average:
Day 1-2	181,677	30	29,27082066	1,024305978	103%
Day 1-3	181,3668	30	29,2204549	1,025984837	Variance:
Day 1-4	183,1679	30	29,5128913	1,016236957	7,6584E-05
Day 1-5	180,3336	30	29,05269893	1,031576702	
Day 1-6	179,1386	30	28,85867222	1,038044259	
Day 2-1	183,9416	30	29,63851344	1,012049552	Average :
Day 2-2	192,3185	30	30,99863252	0,966712249	99%
Day 2-3	190,7145	30	30,73819835	0,975393388	Variance :
Day 2-4	182,3381	30	29,37816046	1,020727985	0,000459909
Day 2-5	189,8807	30	30,60281805	0,979906065	
Day 2-6	187,0245	30	30,13906987	0,995364338	
Day 3-1	182,9344	30	29,47497897	1,017500701	Average:
Day 3-2	184,4544	30	29,72177444	1,009274185	101%
Day 3-3	184,0031	30	29,64849892	1,011716703	Variance:
Day 3-4	187,396	30	30,19938863	0,993353712	0,000192212
Day 3-5	179,7289	30	28,95451654	1,034849449	
Day 3-6	185,3032	30	29,85959023	1,004680326	

6,158946032	1,399595238	**Average recovery:**	101%

Cochran's C test: study of the homogeneity of a series of v

m : number of days for 1 test: 6

p: number of tests for validation: 3

s^2 max	0,000459909
sum s^2	0,000728705
Cochran's critique C, α = 5%	0,7071
C test	**0,631131657**

C test < C 5% : The variances are homogeneous (No rejection of H0)

Estimation of repeatability

Average calculation:	101%
s^2 intra :	0,000242902
**SD repeatability:**	0,015500838
	1,55%

Estimation of intermediate precision

Average calculation:	101%
Average s^2 :	3,86616E-08
s^2IG :	2,3197E-07
s^2 facteur :	-4,0445E-05
s^2RI:	0,000202457
**SD intermediate precision:**	0,014151615
	1,42%

Precision

CLONIDINE within the mixture

Essay Number	AUC (mAu*min)	Concentration (µg/mL)	Observed concentration (µg/mL)	Recovery Rate	
Day 1-1	182,5687	30	28,77163949	1,04094535	Average:
Day 1-2	191,0772	30	30,14105531	0,995298156	100%
Day 1-3	188,7007	30	29,75856524	1,008047825	Variance:
Day 1-4	195,1747	30	30,8005348	0,973315507	0,000577868
Day 1-5	192,6216	30	30,38962154	0,987012615	
Day 1-6	193,2537	30	30,49135601	0,983621466	
Day 2-1	192,2494	30	30,32971714	0,989009429	Average :
Day 2-2	188,8567	30	29,78367294	1,007210902	100%
Day 2-3	193,123	30	30,47032026	0,984322658	Variance :
Day 2-4	191,8215	30	30,26084801	0,991305066	0,000122519
Day 2-5	189,445	30	29,87835794	1,004054735	
Day 2-6	188,0835	30	29,65922888	1,011359037	
Day 3-1	184,2875	30	29,04827484	1,031724172	Average:
Day 3-2	188,6166	30	29,74502962	1,008499013	103%
Day 3-3	187,8153	30	29,61606295	1,012797902	Variance:
Day 3-4	174,2105	30	27,42641398	1,085786201	0,000976677
Day 3-5	188,3177	30	29,69692262	1,010102579	
Day 3-6	189,7109	30	29,9211537	1,00262821	

6,213233333	3,803790476	**Average recovery:**	101%

Cochran's C test: study of the homogeneity of

m : number of days for 1 test: 6

p: number of tests for validation: 3

s^2 max	0,000976677
sum s^2	0,001677065
Cochran's critique C, α = 5%	0,7071
C test	**0,582373048**

C test < C 5% : The variances are homogeneous (No rejection of H0)

Estimation of repeatability

Average calculation:	101%
s^2 intra :	0,000559022
**SD repeatability:**	0,02356064
	2,36%

Estimation of intermediate precision

Average calculation:	101%
Average s^2 :	1,82663E-07
s^2IG :	1,09598E-06
s^2 facteur :	-9,29876E-05
s^2RI:	0,000466034
**SD intermediate precision:**	0,021512039
	2,15%

Accuracy

CLONIDINE drug product

Essay	Observed AUC (mAu*min)	Concentration (mg/L)	observed concentration (mg/L)	Recovery rate (%)	Variance of recoveries	Average recovery	
Day 1 25%	128,4622	21	20,63057609	98,24083854			
Day 2 25%	127,2393	21	20,43201939	97,29533044			
Day 3 25%	131,7208	21	21,15966013	100,7602863	**3,2079203**	98,7654851	
Day 1 50%	149,0511	24	23,97350196	99,8895915			
Day 2 50%	149,7241	24	24,0827739	100,3448913			
Day 3 50%	151,2029	24	24,32287992	101,345333	**0,55456081**	100,5266053	
Day 1 100%	170,1337	27	27,39658765	101,4688431			
Day 2 100%	166,4311	27	26,79541336	99,24227169			
Day 3 100%	167,1937	27	26,91923324	99,70086386	**1,382279641**	100,1373262	
Day 1 125%	188,9042	30	30,44426819	101,480894			
Day2 125%	187,797	30	30,26449717	100,8816572			
Day 3 125%	188,7895	30	30,42564487	101,4188162	**0,108579683**	101,2604558	
Day 1 150%	202,9627	33	32,72688277	99,17237203			
Day 2 150%	201,0545	33	32,41705703	98,23350615			
Day 3 150%	206,4397	33	33,29142741	100,8831134	**1,804753919**		
Day 1 200%	222,5938	36	35,91429501	99,7619306			
Day 2 200%	222,1724	36	35,84587422	99,57187282			
Day 3 200%	224,9177	36	36,29161607	100,8100446	**0,444622402**	100,0479494	
Day 1 250%	240,1313	39	38,76177897	99,38917684			
Day 2 250%	238,8034	39	38,5461739	98,83634333			
Day 3 250%	243,8024	39	39,35783875	100,9175352	**1,162144019**	99,7143518	
				Sum of variances	8,664860772	100,0753623	Average of recovery averages
						1,199543918	Total standard deviation of the recovery

6,158946032 1,399595238

Cochran's C test C(7,3)

C test	**0,370221794**
C threshold (cochr	**0,561**
s²max	3,2079203
s² sum	8,664860772

C test < C table, no rejection of H0, the variances are homogeneous at the 5% risk.

ANOVA

	Sum of SES	DOF	s²	Fexp	p-value
**Intragroup**	17,32972154	14	1,237837253		
**Intergroup**	11,44839068	5	2,289678135	1,849740852	0,16756191
**Total**	28,77811222	19			

SES : Sum of standard errors squared

Mean Recovery Confidence Interval

**Average R:**	99,98311963
**total variance :**	1,514637485
**Number of DOF:**	n-1 =20
**t 5%, 20 DOF:**	2,085963447
**lower bound :**	99,32026939
**Upper bound :**	100,6459699

So the mean overlap confidence interval is : [99,32 ; 100,65]
The average recovery rate is 99.9831
It is included in the confidence interval: ok.

Accuracy

CLONIDINE within the mixture

Essay	Observed AUC (mAu*min)	Concentration (mg/L)	observed concentration (mg/L)	Recovery rate (%)	Variance of recoveries	Average recovery	
Day 1 25%	134,9275	21	21,10394097	100,494957			
Day 2 25%	134,3452	21	21,01022165	100,0486745			
Day 3 25%	133,5634	21	20,88439345	99,44949261	**0,275197142**	99,99770806	
Day 1 50%	151,8987	24	23,83540124	99,31417185			
Day 2 50%	152,5736	24	23,94402424	99,76676765			
Day 3 50%	154,6131	24	24,27227523	101,1344801	**0,898167088**	100,0718065	
Day 1 100%	168,6953	27	26,5387602	98,29170446			
Day 2 100%	173,8201	27	27,36358035	101,3465939			
Day 3 100%	170,4483	27	26,82089994	99,33666644	**2,410683862**	99,65832159	
Day 1 125%	190,2357	30	30,00561858	100,0187286			
Day2 125%	191,4522	30	30,20141035	100,6713678			
Day 3 125%	187,4468	30	29,55675406	98,52251352	**1,213695408**	99,73753665	
Day 1 150%	206,9355	33	32,69339789	99,07090268			
Day 2 150%	211,0744	33	33,35954058	101,0895169			
Day 3 150%	210,7729	33	33,31101512	100,9424701	**1,266531775**		
Day 1 200%	229,7488	36	36,36512544	101,0142373			
Day 2 200%	228,7038	36	36,19693603	100,5470445			
Day 3 200%	229,1943	36	36,27588044	100,7663345	**0,054635499**	100,7758721	
Day 1 250%	244,5394	39	38,74562512	99,34775672			
Day 2 250%	247,9516	39	39,29480778	100,7559174			
Day 3 250%	241,276	39	38,22039135	98,00100345	**1,897701933**	99,36822585	
				Sum of variances	8,016612707	99,9349118	Average of recovery averages
						1,000354488	Total standard deviation of the recovery

6,213233333 3,803790476

Cochran's C test C(7,3)

C test	**0,30071103**
C threshold (cochran	**0,561**
s²max	2,410683862
s² sum	8,016612707

C test < C table, no rejection of H0, the variances are homogeneous at the 5% risk.

ANOVA

	Sum of SES	DOF	s²	Fexp	p-value
**Intragroup**	16,03322541	14	1,145230387		
**Intergroup**	3,980956631	5	0,796191326	0,695223717	0,635698181
**Total**	20,01418204	19			

SES : Sum of standard errors squared

Mean Recovery Confidence Interval

**Average R:**	99,99672867
**total variance :**	1,053378002
**Number of DOF:**	n-1 =20
**t 5%, 20 DOF:**	2,085963447
**lower bound :**	99,44394757
**Upper bound :**	100,5495098

So the mean overlap confidence interval is : [99,44 ; 100,54]
The average recovery rate is 99.9831
It is included in the confidence interval: ok.
