# Supplementary material for: Therapeutic Treatment Plan Optimization during the COVID-19 Pandemic: A Comprehensive Physicochemical Compatibility Study of Intensive Care Units Selected Drugs
Source: Pharmaceutics. 2022 Feb 28;14(3):550. doi: 10.3390/pharmaceutics14030550 (PMC8952813; doi:10.3390/pharmaceutics14030550)
Supplement: Supplementary file 1 [file pharmaceutics-14-00550-s001.zip › Supplementary materials Figure S1B-Ketamine method Validation.pdf]

Linearity

KETAMINE drug product

Essay	Observed AUC (mAu)	Concentration (µg/L)	Variance s²	Estimated AUC	Adjusted AUC	Confidence interval
D1 25%	1,46400913	1	0,00947754	1,360667954	1,46400913	0,20452703
D2 25%	1,278485634	1		1,360667954	1,278485634	-0,162650616
D3 25%	1,422417631	1		1,360667954	1,422417631	0,12221148
D1 50%	4,6212	2	0,025829936	4,136776219	4,6212	0,958744247
D2 50%	4,317925558	2		4,136776219	4,317925558	0,358520562
D3 50%	4,561803051	2		4,136776219	4,561803051	0,841189154
D1 100%	9,3436	4	0,066226506	9,688992748	9,3436	-0,683581863
D2 100%	9,626704715	4		9,688992748	9,626704715	-0,123276965
D3 100%	9,8574	4		9,688992748	9,8574	0,333302142
D1 125%	12,2109	5	0,117836544	12,46510101	12,2109	-0,50310032
D2 125%	12,60617335	5		12,46510101	12,60617335	0,279202415
D3 125%	11,9224	5		12,46510101	11,9224	-1,074083261
D1 150%	14,8235	6	0,074742023	15,24120928	14,8235	-0,826706662
D2 150%	14,3713	6		15,24120928	14,3713	-1,721675418
D3 150%	14,3312	6		15,24120928	14,3312	-1,801039078
D1 200%	21,17282967	8	0,112430365	20,79342581	21,17282967	0,75089475
D2 200%	21,83151003	8		20,79342581	21,83151003	2,054517795
D3 200%	21,3931	8		20,79342581	21,3931	1,186841367
D1 250%	26,53069975	10	0,253484928	26,34564234	26,53069975	0,366255196
D2 250%	26,67098278	10		26,34564234	26,67098278	0,64389548
D3 250%	25,73730477	10		26,34564234	25,73730477	-1,203987436

Linear regression (LINEST function)

Slope b	2,776108265	-1,41544031	Intercept a
S slope	0,037394375	0,221679131	S Intercept
R²	0,99656443	0,505269035	S res
F	5511,378579	19	DOF
Regression sum of squares	1407,037304	4,850639164	esidual sum of squares

CHECKING THE FIT TO THE LINEAR MODEL n=3 k=7

Cochran's C test: Study of the homogeneity of a series of variances

k : number of samples per test
C test = max s²/sum s²

C test	**0,384051872**
Cochran's critical C, α = 5%	0,561
Maximum S²	0,253484928
Sum s²	0,660027841

The variances are homogeneous at the risk of 0.05% because 0,384<0,561

Calculation of experimental variance s²exp

s²exp = 0,094289692

Calculation of misfit variance

SCE aj = 3,907742248
s² aj = 1,302580749

Fisher Test

H0: not significantly different
H1: significantly different
Fexp 13,81466762 **F threshold limit 5% (5, 14)** 2,958248913 **p-value** **0,000181489**

Conclusion p-value > 5%, therefore no rejection of H0: Fit to the validated linear model.

DEPENDENCE VERIFICATION F(1, n - 2)

H0: The variances are not significantly different
H1: The variances are significantly different
F exp **5511,378579** **F threshold limit 5%(1, 19)** 4,38 **p-value** **7,07E-25**

Conclusion Dependence is validated as H0 is rejeted, p-value<5%

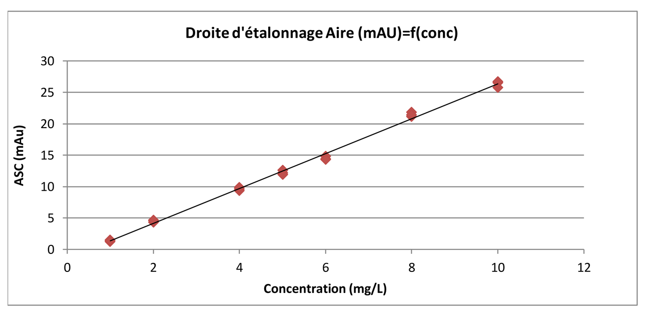

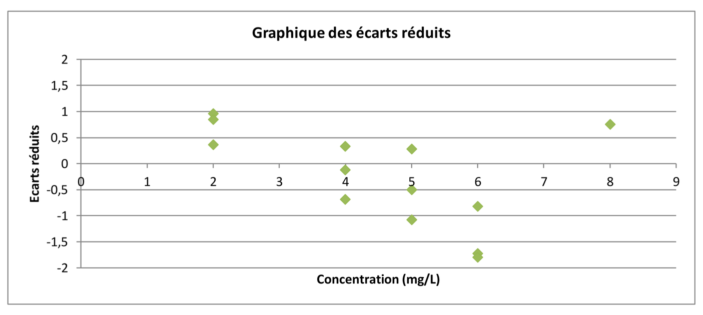

VALIDATION OF THE PROPORTIONAL MODEL

Regression of KETAMINE within the mixture: Comparison of the y-intercept to 0

IaI	1,41544031
Standard deviation of the intercept Sa	0,221679131

Student Test

H0:	The y-intercept is equal to 0
H1:	The y-intercept is different from 0
t exp	**6,385085971**
Nb of DOF (N-2)	19
t threshold (5%, student)	2,093

Conclusion texp > threshold→ Rejection of H0 at 5% risk. The y-intercept is significantly different from 0

the correlation line does not pass through the origin

KETAMINE Pure drug Product Regression: Comparing the Intercept to 0

IaI	0,872160855
standard deviation	0,246711032

Student Test

H0:	The y-intercept is equal to 0
H1:	The y-intercept is different from 0
t exp	**3,535151414**
Nb of DOF (N-2)	19
t threshold (5%, stude	2,093

Conclusion texp > threshold→ Rejection of H0 at 5% risk. The y-intercept is significantly different from 0

the correlation line does not pass through the origin

CALCULATED LIMIT OF DETECTION	**1,82006243**
CALCULATED LIMIT OF QUANTIFICATION	**0,600620602**

COMPARISON OF CLONIDINE IN THE MIXTURE AND THE PUR DRUG PRODUCT REGRESSIONS

Comparison of Student slopes

H0:	No difference between the two slopes
H1:	Difference between the two slopes
texp	**4,612656913**
Nb of DOF (N1+N2-4)	38
t threshold (5%, student à 38 dof)	2,042

Conclusion texp > t threshold→ Rejection of

COMPARISON OF STUDENT INTERCEPTS

H0:	No difference between the two intercepts
H1:	Difference between the two intercepts
t exp	**1,637989847**
Nb of DOF (N1+N2-4)	38
t threshold (5%, student à 38 ddl)	2,042

Conclusion [illegible] of H0 at 5% risk, therefore no

SIGNIFICANCE TEST OF KETAMINE IN THE MIXTURE FORM CORRELATION COEFFICIENT (Pearson correlation coefficient test)

R reconstituted form: 0,998280737

H0 :	R=0
H1 :	R different from 0
texp	**74,2386596**
Nb of DOF:	19
t threshold (5%, student)	2,093

Conclusion texp > t threshold→ Rejection of H0 at 5% risk, existence of a significant linear link between the variables

SIGNIFICANCE TEST OF KETAMINE PURE PRODUCT CORRELATION COEFFICIENT (Pearson correlation coefficient test)

R pure product 0,997415039

H0 :	R=0
H1 :	R different from 0
texp	**60,50504009**
Nb of DOF:	19
t threshold (5%, student)	2,093

Conclusion texp > t threshold→ Rejection of H0 at 5% risk, existence of a significant linear link between the variables

Linearity

KETAMINE within the mixture

Essay	Observed AUC (mAu)	Concentration (µg/L)	Variance s²	Estimated AUC	Adjusted AUC	Confidence interval
D1 25%	1,476925796	1	0,017397656	1,645873146	1,476925796	-0,300444972
D2 25%	1,494472837	1		1,645873146	1,494472837	-0,269240456
D3 25%	1,257747468	1		1,645873146	1,257747468	-0,690217446
D1 50%	3,914898234	2	0,007603481	4,163907148	3,914898234	-0,442821245
D2 50%	3,996755732	2		4,163907148	3,996755732	-0,297251199
D3 50%	3,822466737	2		4,163907148	3,822466737	-0,607195405
D1 100%	9,554731592	4	0,279440979	9,19997515	9,554731592	0,630875767
D2 100%	10,21598237	4		9,19997515	10,21598237	1,80680111
D3 100%	9,170945685	4		9,19997515	9,170945685	-0,051624112
D1 125%	11,37484555	5	0,140366747	11,71800915	8,856811545	-0,610259823
D2 125%	11,82368674	5		11,71800915	11,82368674	0,18793015
D3 125%	11,07964472	5		11,71800915	11,07964472	-1,135225759
D1 150%	15,15411853	6	0,194004473	14,23604315	15,15411853	1,632645506
D2 150%	15,30818545	6		14,23604315	15,30818545	1,906628077
D3 150%	14,48001222	6		14,23604315	14,48001222	0,433858706
D1 200%	18,9967	8	0,052977507	19,27211116	18,9967	-0,489773275
D2 200%	19,45221651	8		19,27211116	19,45221651	0,320287637
D3 200%	19,282	8		19,27211116	19,282	0,017585677
D1 250%	23,0973608	10	0,651955534	24,30817916	23,0973608	-2,153240566
D2 250%	24,71053846	10		24,30817916	24,71053846	0,715529593
D3 250%	23,96805877	10		24,30817916	23,96805877	-0,604847964

Linear regression (LINEST function)

Slope b	2,518034001	-0,872160855	Intercept a
S slope	0,04161693	0,246711032	S Intercept
R²	0,99483676	0,562323773	S res
F	3660,859876	19	DOF
Regression sum of squares	1157,593272	6,00795248	Residual sum of squares

CHECKING THE FIT TO THE LINEAR MODEL n=3 k=7

Cochran's C test: Study of the homogeneity of a series of variances

k : number of samples per test
C test = max s²/sum s²

C test	0,485177519
Cochran's critical C, α = 5%	0,561
Maximum S²	0,651955534
Sum s²	1,343746376

The variances are homogeneous at the risk of 0.05% because 0,4851<0,561

Calculation of experimental variance s²exp

s²exp = 0,191963768

Calculation of misfit variance

SCE aj = 4,0883148
s² aj = 1,3627716

Fisher Test

H0: not significantly different
H1: significantly different
Fexp 7,09910841 **F threshold limit 5%(5, 14)** 2,958248913 **p-value** **0,003918571**

Conclusion p-value > 5%, therefore no rejection of H0: Fit to the validated linear model.

DEPENDENCE VERIFICATION F(1, n - 2)

H0 : The variances are not significantly different
H1 : The variances are significantly different
F exp 3660,859876 **F threshold limit 5% (1,19)** 4,38 **p value** **2,53879E-17**

Conclusion Dependence is validated as H0 is rejeted, p-value<5%

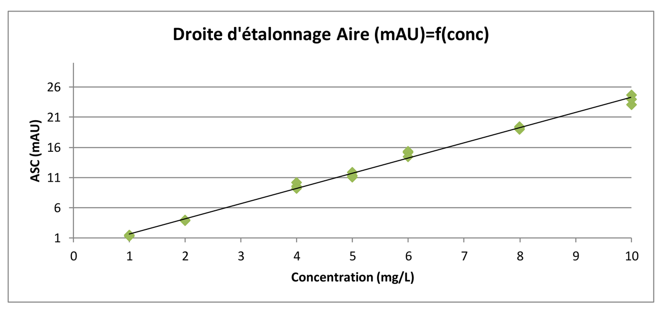

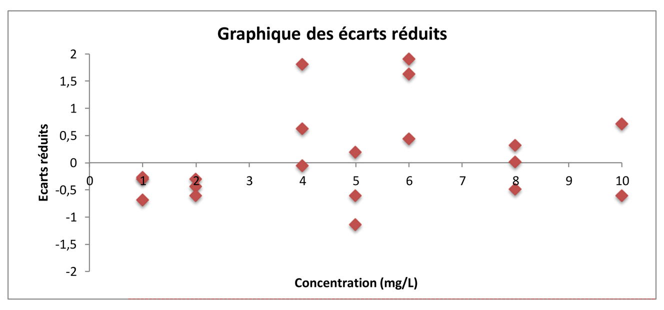

Precision

KETAMINE drug product

Essay Number	AUC (mAu*min)	Concentration (µg/mL)	Observed concentration (µg/mL)	Recovery Rate	
Day 1-1	11,1643	5	4,780102591	104%	Average:
Day 1-2	11,2314	5	4,806750365	104%	103%
Day 1-3	11,0329	5	4,727919023	105%	Variance:
Day 1-4	12,0338	5	5,12541167	97%	0,000857776
Day 1-5	11,0886	5	4,750039455	105%	
Day 1-6	11,2528	5	4,815249059	104%	
Day 2-1	11,8274	5	5,043442959	99%	Average :
Day 2-2	11,9117	5	5,076921459	98%	100%
Day 2-3	11,0395	5	4,730540115	105%	Variance :
Day 2-4	11,6646	5	4,978789344	100%	0,000840733
Day 2-5	11,964	5	5,097691631	98%	
Day 2-6	12,0275	5	5,122909718	98%	
Day 3-1	10,7564	5	4,618111133	108%	Average:
Day 3-2	11,7393	5	5,008455346	100%	102%
Day 3-3	11,101	5	4,754963932	105%	Variance:
Day 3-4	11,4303	5	4,885740561	102%	0,001362522
Day 3-5	11,8952	5	5,070368727	99%	
Day 3-6	11,8938	5	5,069812738	99%	

2,518034001	-0,872160855	**Average recovery:**	102%	

Cochran's C test: study of the homogeneity of a series of variances C (m,p)

s^2 max	0,003061031		*m : number of days for 1 test: 6*
sum s^2	0,7071		*p: number of tests for validation: 3*
Cochran's critique C, α = 5%		0,5685	
	**0**		
C test			

C test < C 5% : The variances are homogeneous (No rejection of H0)

Estimation of repeatability

Average calculation:	102%
s^2 intra :	0,001020344
**SD repeatability:**	0,031673546
	3,17%

Estimation of intermediate precision

Average calculation:	102%
Average s^2 :	8,78873E-08
s^2IG :	5,27324E-07
s^2 facteur :	-0,000169969
s^2RI:	0,000850374
**SD intermediate precision:**	0,028915354
	2,89%

Precision

KETAMINE within the mixture

Essay Number	AUC (mAu*min)	Concentration (µg/mL)	Observed concentration (µg/mL)	Recovery Rate	
Day 1-1	12,7083	5	5,107222876	98%	Average:
Day 1-2	12,63	5	5,159525585	97%	97%
Day 1-3	12,346	5	5,156586785	97%	Variance:
Day 1-4	12,4507	5	5,159724153	97%	0,00010402
Day 1-5	12,4978	5	5,173306178	97%	
Day 1-6	12,0473	5	5,03951132	99%	
Day 2-1	11,988	5	5,107222876	98%	Average :
Day 2-2	12,1197	5	5,159525585	97%	97%
Day 2-3	12,1123	5	5,156586785	97%	Variance :
Day 2-4	12,1202	5	5,159724153	97%	0,00010402
Day 2-5	12,1544	5	5,173306178	97%	
Day 2-6	11,8175	5	5,03951132	99%	
Day 3-1	11,8564	5	5,054959881	99%	Average:
Day 3-2	11,7621	5	5,017510029	100%	100%
Day 3-3	11,991	5	5,108414282	98%	Variance:
Day 3-4	11,4393	5	4,889314778	102%	0,000421534
Day 3-5	11,3155	5	4,840149437	103%	
Day 3-6	11,5691	5	4,94086293	101%	

2,518034001	-0,872160855	**Average recovery:**	98%	

Cochran's C test: study of the homogeneity of a series of variances C (m,p)

s^2 max	0,000629575		*m : number of days for 1 test: 6*
sum s^2	0,7071		*p: number of tests for validation: 3*
Cochran's critique C, α = 5%		0,5685	
	**0**		
C test			

C test < C 5% : The variances are homogeneous (No rejection of H0)

Estimation of repeatability

Average calculation:	98%
s^2 intra :	0,000209858
**SD repeatability:**	0,014604035
	1,46%

Estimation of intermediate precision

Average calculation:	98%
Average s^2 :	3,3605E-08
s^2IG :	2,0163E-07
s^2 facteur :	-3,49428E-05
s^2RI:	0,000174916
**SD intermediate precision:**	0,01333288
	1,33%

Accuracy

ketamine drug product

Essai	Observed AUC (mAu*min)	Concentration (mg/L)	observed concentration (mg/L)	Recovery rate (%)	Variance of recoveries	Average recovery	
Day 1 25%	1,476925796	1	0,932905056	93,2905056			
Day 2 25%	1,494472837	1	0,939873604	93,98736042			
Day 3 25%	1,577474682	1	0,972836561	97,28365606	**4,549403694**	93,63893301	
Day 1 50%	3,914898234	2	1,901109789	95,05548946			
Day 2 50%	3,996755732	2	1,933618285	96,68091425			
Day 3 50%	3,822466737	2	1,864401986	93,22009928	**2,997983862**	94,985501	
Day 1 100%	9,554731592	4	4,140886279	103,522157			
Day 2 100%	10,21598237	4	4,403492255	110,0873064			
Day 3 100%	9,170945685	4	3,988471377	99,71178442	**27,54526354**	104,4404159	
Day 1 125%	11,37484555	5	4,863717644	97,27435288			
Day2 125%	11,82368674	5	5,041968294	100,8393659			
Day 3 125%	11,07964472	5	4,746482998	94,92965995	**8,855254452**	97,68112624	
Day 1 150%	15,15411853	6	6,364600073	106,0766679			
Day 2 150%	15,30818545	6	6,425785471	107,0964245			
Day 3 150%	14,48001222	6	6,096888709	101,6148118	**8,49935681**	104,9293014	
Day 1 200%	18,9967	8	7,890624529	98,63280661			
Day 2 200%	19,45221651	8	8,07152618	100,8940772			
Day 3 200%	19,282	8	8,003927208	100,0490901	**1,30553452**	99,85865799	
Day 1 250%	23,0973608	10	9,519141379	95,19141379			
Day 2 250%	24,71053846	10	10,15979105	101,5979105			
Day 3 250%	23,96805877	10	9,864926213	98,64926213	**10,28240713**	98,47952881	
				Sum of variances	64,035204	99,14478062	Average of recovery averages
						4,628011726	Total standard deviation of the recovery

2,518034001 -0,872160855

Cochran's C test C(7,3)

C test	**0,430158129**
C threshold (cochran table) 5%	0,561
s^2max	27,54526354
s^2 sum	64,035204

C test < C table, no rejection of H0, the variances are homogeneous at the 5% risk.

ANOVA

	Sum of SES	DOF	s^2	Fexp	p-value
Intragroup	128,070408	14	9,147886286		
Intergroup	300,2994427	5	60,05988853	6,565438907	0,002413872
Total	428,3698507	19			

SES : Sum of standard errors squared

Mean Recovery Confidence Interval

**Average R:**	99,31833886
**total variance :**	22,54578161
**Number of DOF:**	n-1 =20
**t 5%, 20 DOF:**	2,085963447
**lower bound :**	96,760968
**Upper bound :**	101,8757097

So the mean overlap confidence interval is : [95,6035 ; 101,8239]
The average recovery rate is 98,7137
It is included in the confidence interval: ok.

Accuracy

ketamine within the mixture

Essay	Observed AUC (mAu*min)	Concentration (mg/L)	served concentration (mg	Recovery rate (%)	Variance of recoveries	Average recovery	
Day 1 25%	1,46400913	1	1,037225196	103,7225196			
Day 2 25%	1,278485634	1	0,970396572	97,03965724			
Day 3 25%	1,422417631	1	1,022243252	102,2243252	**1,122293251**	100,3810884	
Day 1 50%	4,6212	2	2,174497438	108,7248719			
Day 2 50%	4,317925558	2	2,065252981	103,262649			
Day 3 50%	4,561803051	2	2,153101678	107,6550839	**8,378968221**	106,5475349	
Day 1 100%	9,3436	4	3,875583833	96,88959583			
Day 2 100%	9,626704715	4	3,977562823	99,43907057			
Day 3 100%	9,8574	4	4,060663071	101,5165768	**5,37080099**	99,28174772	
Day 1 125%	12,2109	5	4,908432601	98,16865202			
Day2 125%	12,60617335	5	5,050816583	101,0163317			
Day 3 125%	11,9224	5	4,804510141	96,09020282	**6,11599594**	98,42506216	
Day 1 150%	14,8235	6	5,849534226	97,49223711			
Day 2 150%	14,3713	6	5,686644325	94,77740542			
Day 3 150%	14,3312	6	5,672199644	94,53666073	**2,693950128**		
Day 1 200%	21,17282967	8	8,136667532	101,7083442			
Day 2 200%	21,83151003	8	8,373935065	104,6741883			
Day 3 200%	21,3931	8	8,216012538	102,7001567	**2,279454075**	103,0275631	
Day 1 250%	26,53069975	10	10,06666073	100,6666073			
Day 2 250%	26,67098278	10	10,117193	101,17193			
Day 3 250%	25,73730477	10	9,78086677	97,8086677	**3,289117157**	99,88240167	
				Sum of variances	29,25057976	101,2575663	Average of recovery averages
						3,878942378	Total standard deviation of the recovery

2,776108265 -1,41544031

Cochran's C test C(7,3)

C test	**0,183613488**
C threshold (cochran	0,561
s^2max	5,37080099
s^2 sum	29,25057976

C test < C table, no rejection of H0, the variances are homogeneous at the 5% risk.

ANOVA

	Sum of SES	DOF	s^2	Fexp	p-value
Intragroup	58,50115952	14	4,178654252		
Intergroup	242,4227199	5	48,48454399	11,60290875	0,000141301
Total	300,9238795	19			

SES : Sum of standard errors squared

Mean Recovery Confidence Interval

**Average R:**	100,5374159
**total variance :**	15,83809892
**Number of DOF:**	n-1 =20
**t 5%, 20 DOF:**	2,085963447
**lower bound :**	98,39396969
**Upper bound :**	102,6808621

So the mean overlap confidence interval is : [98,3940 ; 102,6809]
The average recovery rate is 100,5374
It is included in the confidence interval: ok.
