# Supplementary material for: Therapeutic Treatment Plan Optimization during the COVID-19 Pandemic: A Comprehensive Physicochemical Compatibility Study of Intensive Care Units Selected Drugs
Source: Pharmaceutics. 2022 Feb 28;14(3):550. doi: 10.3390/pharmaceutics14030550 (PMC8952813; doi:10.3390/pharmaceutics14030550)
Supplement: Supplementary file 1 [file pharmaceutics-14-00550-s001.zip › Supplementary materials Figure S1C-Loxapine method Validation.pdf]

LINEARITY

LOXAPINE within the mixture

Essay	Observed AUC (mAu)	Concentration (µg/L)	Variance s²	Estimated AUC	Adjusted AUC	Confidence interval
D1 25%	6,035305681	5	0,405964319	1,044590036	6,035305681	0,760428901
D2 25%	6,016583598	5		1,044590036	6,016583598	0,757576242
D3 25%	7,129407403	5		1,044590036	7,129407403	0,927135769
D1 50%	28,79663711	10	3,047761949	22,09685315	28,79663711	1,020837434
D2 50%	29,31139338	10		22,09685315	29,31139338	1,099270184
D3 50%	26,0632703	10		22,09685315	26,0632703	0,604357862
D1 100%	58,39875211	20	0,079454578	64,20137938	58,39875211	-0,884138827
D2 100%	58,03962295	20		64,20137938	58,03962295	-0,938858873
D3 100%	57,84284537	20		64,20137938	57,84284537	-0,968841619
D1 125%	81,19942166	25	17,40136048	85,25364249	81,19942166	-0,617736395
D2 125%	78,86340892	25		85,25364249	78,86340892	-0,97367164
D3 125%	73,09517965	25		85,25364249	73,09517965	-1,852569289
D1 150%	103,8483443	30	6,045077926	106,3059056	103,8483443	-0,374455451
D2 150%	99,80873165	30		106,3059056	99,80873165	-0,98996601
D3 150%	99,40029141	30		106,3059056	99,40029141	-1,052199522
D1 200%	151,2165161	40	11,37442323	148,4104318	151,2165161	0,427559438
D2 200%	148,9022385	40		148,4104318	148,9022385	0,074935946
D3 200%	144,5724511	40		148,4104318	144,5724511	-0,58478817
D1 250%	198,9540936	50	0,313521196	190,5149581	198,9540936	1,285860184
D2 250%	198,0750972	50		190,5149581	198,0750972	1,151928651
D3 250%	197,9136897	50		190,5149581	197,9136897	1,127335186

Linear regression (LINEST function)

Slope b	4,210452623	-20,00767308	Intercept a
S slope	0,097144407	2,879428759	S Intercept
R²	0,989987071	6,563027309	S res
F	1878,546586	19	DOF
Regression sum of squares	80915,25223	818,3932216	Residual sum of squares

CHECKING THE FIT TO THE LINEAR MODEL n=3 k=7

Cochran's C test: Study of the homogeneity of a series of variances

k : nombre d'échantillons par essai
C test = s²max/somme s²

C test	0,450024745
Cochran's critical C, α = 5%	0,561
Maximum S²	17,40136048
Sum s²	38,66756368

The variances are homogeneous at the risk of 0.05% because 0,4835<0,5685

75,124
76,059
74,29
75,94
72,532
75,852

Calculation of experimental variance s²exp

s²exp = 5,523937669

Calculation of misfit variance

SCE aj = 763,153845
s² aj = 254,384615

Fisher Test

H0: Not significantly different
H1: Significantly different
Fexp 46,05131887 **F threshold 5% (5, 14)** 2,958248913 **p-value** **3,34971E-08**

Conclusion p-value > 5%, therefore no rejection of H0: Fit to the validated linear model.

DEPENDENCE VERIFICATION. F(1, n - 2)

H0: The variances are not significantly different
H1: The variances are significantly different
F exp **1878,546586** **F threshold 5% (1, 19)** 4,38 **p-value** **1,84E-20**

Conclusion Dependence is validated as H0 is rejeted, p-value<5%

VALIDATION OF THE PROPORTIONAL MODEL

Regression of LOXAPINE within the mixture: Comparison of the y-intercept to 0

IaI 20,00767308
Standard deviation of the intercept S 2,879428759

Student Test

H0: The y-intercept is equal to 0
H1: The y-intercept is different from 0
t exp **6,948486922**
Nb of DOF (N-2) 19
t threshold (5%, student) 2,093

Conclusion texp > threshold→ Rejection of H0 at 5% risk. The y-intercept is significantly different from 0

the correlation line does not pass through the origin

LOXAPINE Pure drug Product Regression: Comparing the Intercept to 0

IaI 22,23347685
Standard deviation of the in 2,67944703

Student Test

H0: The y-intercept is equal to 0
H1: The y-intercept is different from 0
t exp **8,29778555**
Nb of DOF (N-2) 19
t threshold (5%, student) 2,093

Conclusion texp > threshold→ Rejection of H0 at 5% risk. The y-intercept is significantly different from 0

the correlation line does not pass through the origin

CALCULATED LIMIT OF DETECTION	15,58746267
CALCULATED LIMIT OF QUANTIFICATION	5,143862682

COMPARISON OF CLONIDINE IN THE MIXTURE AND THE PUR DRUG PRODUCT REGRESSIONS

Comparison of Student slopes

H0: No difference between the two slopes
H1: Difference between the two slopes
texp **0,085243682**
Nb of DOF (N1+N2-4) 38
t threshold (5%, student à 38 dof) 2,042

Conclusion texp < t threshold → No rejection of H0 at 5% risk, no significant difference between the 2 slopes.

COMPARISON OF STUDENT INTERCEPTS

H0: No difference between the two intercepts
H1: Difference between the two intercepts
t exp **0,565892644**
Nb of DOF (N1+N2-4) 38
t threshold (5%, student à 38 ddl) 2,042

Conclusion texp < t threshold→ No rejection of H0 at 5% risk therefore no systematic error.

SIGNIFICANCE TEST OF CLONIDINE IN THE MIXTURE FORM CORRELATION COEFFICIENT (Pearson correlation coefficient test)

H0: R=0 R forme reconstituée: 0,99498094
H1: R different from 0
texp **43,34220329**
Nb of DOF 19
t threshold (5%, student) 2,093

Conclusion texp > t threshold→ Rejection of H0 at 5% risk, existence of a significant linear link between the variables

SIGNIFICANCE TEST OF CLONIDINE PURE PRODUCT CORRELATION COEFFICIENT (Pearson correlation coefficient test)

R pure product 0,995626195

H0 : R=0
H1 : R different from 0
texp **46,45193561**
Nb of DOF: 19
t threshold (5%, student) 2,093

Conclusion texp > t threshold → Rejection of H0 at 5% risk, existence of a significant linear link between the variables

LINEARITY

LOXAPINE drug product

Essay	Observed AUC (mAu)	Concentration (µg/L)	Variance s²	Estimated AUC	AdjustedAUC	Confidence interval
D1 25%	6,666106415	5	0,276074245	-1,237772056	6,666106415	1,294187511
D2 25%	5,670646023	5		-1,237772056	5,670646023	1,131190014
D3 25%	5,8768	5		-1,237772056	5,8768	1,164945835
D1 50%	19,97909416	10	1,410007363	19,75793274	19,97909416	0,036213152
D2 50%	20,58284873	10		19,75793274	20,58284873	0,135072418
D3 50%	22,2701	10		19,75793274	22,2701	0,411344318
D1 100%	56,13142117	20	0,01676441	61,74934233	56,13142117	-0,919882996
D2 100%	55,88686705	20		61,74934233	55,88686705	-0,959926487
D3 100%	55,9354	20		61,74934233	55,9354	-0,951979661
D1 125%	77,43903879	25	10,34456119	82,74504712	56,443334	-0,868810134
D2 125%	75,42167693	25		82,74504712	75,42167693	-1,199134612
D3 125%	81,7201	25		82,74504712	81,7201	-0,167825678
D1 150%	98,82472467	30	10,14751669	103,7407519	98,82472467	-0,804954313
D2 150%	95,85890629	30		103,7407519	95,85890629	-1,290579835
D3 150%	102,225	30		103,7407519	102,225	-0,248190455
D1 200%	148,335377	40	34,70564574	145,7321615	148,335377	0,426252631
D2 200%	139,6769	40		145,7321615	139,6769	-0,99149346
D3 200%	150,9264	40		145,7321615	150,9264	0,850508851
D1 250%	196,6446373	50	45,40435749	187,7235711	196,6446373	1,460742659
D2 250%	186,0310594	50		187,7235711	186,0310594	-0,277133248
D3 250%	198,53	50		187,7235711	198,53	1,769453489

Linear regression (LINEST function)

Slope b	4,199140959	-22,23347685	Intercept a
S slope	0,090397545	2,67944703	S Intercept
R²	0,99127152	6,107212751	S res
F	2157,782322	19	DOF
Regression sum of squares	80481,06773	708,662904	Residual sum of squares

CHECKING THE FIT TO THE LINEAR MODEL n=3 k=7

Cochran's C test: Study of the homogeneity of a series of variances

p=5, n=3

C test	0,443813986
Cochran's critical C, α = 5%	0,684
Maximum S²	45,40435749
Sum s²	102,3049271

The variances are homogeneous at the risk of 0.05% because 0,341<0,684

Calculation of experimental variance s²exp

s²exp = 14,61498959

Calculation of misfit variance

SCE aj = 562,5130081
s² aj = 187,504336

Fisher Test

H0: Not significantly different
H1: Significantly different
Fexp **12,8295908** **F threshold 5%(5,14)** 2,958248913 **p-value** **8,16657E-05**

Conclusion p-value > 5%, therefore no rejection of H0: Fit to the validated linear model.

DEPENDENCE VERIFICATION. F(1, n - 2)

H0: The variances are not significantly different
H1: The variances are significantly different
F exp 2157,782322 **F threshold 5% (1,19)** 4,38 **p value** **4,98299E-21**

Conclusion Dependence is validated as H0 is rejeted, p-value<5%

Precision

LOXAPINE PUR DRUG

Essay Number	AUC (mAu*min)	Concentration (µg/mL)	Observed concentration (µg/mL)	Recovery Rate	
Day 1-1	85,20762243	25	25,5864474	98%	Average:
Day 1-2	81,62173369	25	24,73248971	101%	99%
Day 1-3	83,93600088	25	25,28361843	99%	Variance:
Day 1-4	83,82742447	25	25,25776161	99%	0,000196917
Day 1-5	85,59548421	25	25,67881434	97%	
Day 1-6	82,85592282	25	25,02640438	100%	
Day 2-1	82,97827174	25	25,05554103	100%	Average :
Day 2-2	80,01471794	25	24,34978863	103%	101%
Day 2-3	83,32224931	25	25,13745721	99%	Variance :
Day 2-4	80,32200797	25	24,42296789	102%	0,000259836
Day 2-5	84,00429621	25	25,29988255	99%	
Day 2-6	81,07740362	25	24,6028608	102%	
Day 3-1	84,26344762	25	25,36159789	99%	Average:
Day 3-2	83,4972141	25	25,17912401	99%	100%
Day 3-3	82,467397	25	24,9338793	100%	Variance:
Day 3-4	83,06564665	25	25,07634884	100%	5,80727E-05
Day 3-5	82,13577351	25	24,85490518	101%	
Day 3-6	82,43004033	25	24,92498304	100%	

4,199140959	-22,23347685	**Average recovery:**	100%

Cochran's C test: study of the homogeneity of a series of variances C (m,p)

m : number of days for 1 test: 6

p: number of tests for validation: 3

s^2 max	0,000259836
sum s^2	0,000514825
Cochran's critique C, α = 5%	0,7071
C test	**0,504706567**
C test < C 5% :	The variances are homogeneous (No rejection of H0)

Estimation of repeatability

Average calculation:	100%
s^2 intra :	0,000171608
**SD repeatability:**	0,013111087
	1,31%

Estimation of intermediate precision

Average calculation:	100%
Average s^2 :	8,10143E-05
s^2IG :	0,000486086
s^2 facteur :	5,24129E-05
s^2RI:	0,000224021
**SD intermediate precision:**	0,014980078
	1,50%

Precision

LOXAPINE within the mixture

Essay Number	AUC (mAu*min)	Concentration (µg/mL)	Observed concentration (µg/mL)	Recovery Rate	
Day 1-1	84,20762243	25	25,34830346	99%	Average:
Day 1-2	83,25688062	25	25,12189005	100%	100%
Day 1-3	83,23485982	25	25,11664593	100%	Variance:
Day 1-4	83,82742447	25	25,25776161	99%	0,000131198
Day 1-5	81,90345761	25	24,79958055	101%	
Day 1-6	81,04857639	25	24,59599577	102%	
Day 2-1	81,92570238	25	24,80487801	101%	Average :
Day 2-2	82,94672905	25	25,04802933	100%	100%
Day 2-3	83,12694675	25	25,09094709	100%	Variance :
Day 2-4	80,32200797	25	24,42296789	102%	0,000156692
Day 2-5	84,12900063	25	25,32958015	99%	
Day 2-6	83,10474791	25	25,08566057	100%	
Day 3-1	84,26344762	25	25,36159789	99%	Average:
Day 3-2	81,12489362	25	24,61417025	102%	100%
Day 3-3	82,09378356	25	24,84490553	101%	Variance:
Day 3-4	82,06515852	25	24,83808865	101%	0,000209924
Day 3-5	83,49954091	25	25,17967813	99%	
Day 3-6	85,12490245	25	25,56674814	98%	

4,199140959	-22,23347685	**Average recovery:**	100%

Cochran's C test: study of the homogeneity of a series of varia

m : number of days for 1 test: 6

p: number of tests for validation: 3

s^2 max	0,000156692
sum s^2	0,000497813
Cochran's critique C, α = 5%	0,7071
C test	**0,314759984**
C test < C 5% :	The variances are homogeneous (No rejection of H0)

Estimation of repeatability

Average calculation:	100%
s^2 intra :	0,000165938
**SD repeatability:**	0,012887805
	2,40%

Estimation of intermediate precision

Average calculation:	100%
Average s^2 :	4,63247E-06
s^2IG :	2,77948E-05
s^2 facteur :	-2,30238E-05
s^2RI:	0,000142914
**SD intermediate precision:**	0,011960343
	3,10%
