# Supplementary material for: Therapeutic Treatment Plan Optimization during the COVID-19 Pandemic: A Comprehensive Physicochemical Compatibility Study of Intensive Care Units Selected Drugs
Source: Pharmaceutics. 2022 Feb 28;14(3):550. doi: 10.3390/pharmaceutics14030550 (PMC8952813; doi:10.3390/pharmaceutics14030550)
Supplement: Supplementary file 1 [file pharmaceutics-14-00550-s001.zip › Supplementary materials Figure S1D-Midazolam method Validation.pdf]

Linearity

MIDAZOLAM drug product

Essay	Observed AUC (mAu)	Concentration (µg/L)	Variance s²	Estimated AUC	Adjusted AUC	Confidence interval
D1 25%	8,1053	5	0,486363773	4,941746889	8,1053	1,383407702
D2 25%	8,3127	5		4,941746889	8,3127	1,474102799
D3 25%	7,0145	5		4,941746889	7,0145	0,906405715
D1 50%	9,6464	6	0,182799543	8,759282901	9,6464	0,387932361
D2 50%	10,0161	6		8,759282901	10,0161	0,549600526
D3 50%	10,499	6		8,759282901	10,499	0,760770548
D1 100%	15,5306	8	0,262072243	16,39435493	15,5306	-0,377716187
D2 100%	16,4835	8		16,39435493	16,4835	0,038982744
D3 100%	16,3314	8		16,39435493	16,3314	-0,027529909
D1 125%	19,9329	10	4,66485841	24,02942695	19,9329	-1,791393011
D2 125%	21,3962	10		24,02942695	21,3962	-1,151498431
D3 125%	24,1843	10		24,02942695	24,1843	0,067725295
D1 150%	59,5402	20	4,23363828	62,20478708	59,5402	-1,165212074
D2 150%	63,6142	20		62,20478708	63,6142	0,616330001
D3 150%	61,0744	20		62,20478708	61,0744	-0,49431324
D1 200%	77,2558	25	1,758809443	81,29246714	77,2558	-1,765216582
D2 200%	79,6734	25		81,29246714	79,6734	-0,708010857
D3 200%	79,4095	25		81,29246714	79,4095	-0,823413153
D1 250%	177,0261	50	1,573380063	176,7308675	177,0261	0,12910388
D2 250%	179,5224	50		176,7308675	179,5224	1,220724765
D3 250%	178,4899	50		176,7308675	178,4899	0,769217108

Linear regression (LINEST function)

Slope b	3,817536013	-14,14593317	Intercept a
S slope	0,033497984	0,775327669	S Intercept
R²	0,998539203	2,286782925	S res
F	12987,60127	19	DOF
Regression sum of squares	67917,0523	99,35814681	Residual sum of squares

CHECKING THE FIT TO THE LINEAR MODEL n=3 k=7

Cochran's C test: Study of the homogeneity of a series of variances

k : number of samples per test
C test = max s²/sum s²

C test	**0,354420767**
Cochran's critical C, α = 5%	0,561
Maximum S²	4,66485841
Sum s²	13,16192176

The variances are homogeneous at the risk of 0.05% because 0,354<0,561

Calculation of experimental variance s²exp

s²exp = 1,880274537

Calculation of misfit variance

SCE aj = 80,55540144
s² aj = 26,85180048

Fisher Test

H0:	Not significantly different				
H1:	Significantly different				
Fexp	14,28078717	F THRESHOLD 5% (5, 14)	2,958248913	p-value	4,49065E-05
**Conclusion**	p-value > 5%, therefore no rejection of H0: Fit to the validated linear model.				

DEPENDENCE VERIFICATION. F(1, n - 2)

H0:	The variances are not significantly different				
H1:	The variances are significantly different				
F exp	**12987,60127**	F threshold 5% (1, 19)	**4,38**	p-value	2,09287E-28
**Conclusion**	Dependence is validated as H0 is rejeted, p-value<5%				

Linearity

MIDAZOLAM within the mixture

<

Essay	Observed AUC (mAu)	Concentration (µg/L)	Variance s²	Estimated AUC	Adjusted AUC	Confidence interval
D1 25%	11,9606	5	0,081154943	12,65507912	11,9606	-1,01643855
D2 25%	12,5097	5		12,65507912	12,5097	-0,212776647
D3 25%	12,3668	5		12,65507912	12,3668	-0,421924864
D1 50%	16,7904	6	0,154529053	16,91787065	16,7904	-0,186565838
D2 50%	16,1378	6		16,91787065	16,1378	-1,141710181
D3 50%	16,8438	6		16,91787065	16,8438	-0,108409681
D1 100%	25,0072	8	0,410336143	25,44345371	25,0072	-0,638500244
D2 100%	26,1641	8		25,44345371	26,1641	1,054736792
D3 100%	25,109	8		25,44345371	25,109	-0,489505923
D1 125%	34,2853	10	0,02863164	33,96903677	34,2853	0,462882375
D2 125%	34,1191	10		33,96903677	34,1191	0,219632314
D3 125%	34,4575	10		33,96903677	34,4575	0,714914027
D1 150%	75,9421	20	0,278535303	76,59695207	75,9421	-0,958440467
D2 150%	76,9176	20		76,59695207	76,9176	0,469299812
D3 150%	76,0807	20		76,59695207	76,0807	-0,755585724
D1 200%	99,4188	25	0,693013463	97,91090972	99,4188	2,206945862
D2 200%	98,0661	25		97,91090972	98,0661	0,227136251
D3 200%	99,5831	25		97,91090972	99,5831	2,447415086
D1 250%	204,1925	50	0,01606336	204,480698	204,1925	-0,42180611
D2 250%	204,0261	50		204,480698	204,0261	-0,66534889
D3 250%	203,9437	50		204,480698	203,9437	-0,785949402

Linear regression (LINEST function)

Slope b	4,26279153	-8,658878536	Intercept a
S slope	0,010008564	0,231653253	S Intercept
R²	0,999895272	0,683247517	S res
F	181403,0598	19	DOF
Regression sum of squares	84683,87682	8,869716209	Residual sum of squares

CHECKING THE FIT TO THE LINEAR MODEL n=3 k=7

Cochran's C test: Study of the homogeneity of a series of variances

k : number of samples per test
C test = max s²/sum s²

C test	0,416909409
Cochran's critical C, α = 5%	0,561
Maximum S²	0,693013463
Sum s²	1,662263907

The variances are homogeneous at the risk of 0.05% because 0,417<0,561

Calculation of experimental variance s²exp

s²exp = 0,237466272

Calculation of misfit variance

SCE aj = 6,495053485
s² aj = 2,165017828

Fisher Test

H0:	Not significantly different				
H1:	Significantly different				
Fexp	9,117159277	F THRESHOLD 5% (5, 14)	2,958248913	p-value	0,000498523
**Conclusion**	p-value > 5%, therefore no rejection of H0: Fit to the validated linear model.				

DEPENDENCE VERIFICATION. F(1, n - 2)

H0:	The variances are not significantly different				
H1:	The variances are significantly different				
F exp	**181403,0598**	F threshold 5% (1, 19)	**4,38**	p-value	2,80205E-39
**Conclusion**	Dependence is validated as H0 is rejeted, p-value<5%				

VALIDATION OF THE PROPORTIONAL MODEL

Regression of midazolam within the mixture: Comparison of the y-intercept to 0

IaI	14,14593317
Standard deviation of the intercept Sa	0,775327669

Student Test

H0:	L'ordonnée à l'origine est égale à 0
H1:	L'ordonnée à l'origine est différente de 0
t exp	**18,24510299**
Nb of DOF (N-2)	19
t threshold (5%, student)	2,093
**Conclusion**	texp > threshold→ Rejection of H0 at 5% risk. The y-intercept is significantly different from 0

the correlation line does not pass through the origin

Pure drug Product Regression: Comparing the Intercept to 0

IaI	8,658878536
standard deviation	0,231653253

Student Test

H0:	L'ordonnée à l'origine est égale à 0
H1:	L'ordonnée à l'origine est différente de 0
t exp	**37,37861829**
Nb of DOF (N-2)	19
t threshold (5%, student)	2,093
**Conclusion**	texp > threshold→ Rejection of H0 at 5% risk. The y-intercept is significantly different from 0

the correlation line does not pass through the origin

CALCULATED LIMIT OF DETECTION	**5,990206557**
CALCULATED LIMIT OF QUANTIFICATION	**1,976768164**

COMPARISON OF CLONIDINE IN THE MIXTURE AND THE PUR DRUG PRODUCT REGRESSIONS

Comparison of Student slopes

H0:	No difference between the two slopes
H1:	Difference between the two slopes
texp	**12,73570059**
Nb of DOF (N1+N2-4)	38
t threshold (5%, student à 38 dof)	2,042
**Conclusion**	texp > t threshold→ Rejection of H0 a 5% risk, significant difference between the 2 slopes

COMPARISON OF STUDENT INTERCEPTS

H0:	No difference between the two intercepts
H1:	Difference between the two intercepts
t exp	**6,780882227**
Nb of DOF (N1+N2-4)	38
t threshold (5%, student à 38 ddl)	2,042
**Conclusion**	texp > t threshold→ rejection of H0 at 5 % risk, therefore systematic error

SIGNIFICANCE TEST OF MIDAZOLAM IN THE MIXTURE FORM CORRELATION COEFFICIENT (Pearson correlation coefficient test)

R reconstituted form: 0,999269335

H0 :	R=0
H1 :	R different from 0
texp	**113,9631575**
Nb of DOF:	19
t threshold (5%, student)	2,093
**Conclusion**	texp > t threshold→ Rejection of H0 at 5% risk, existence of a significant linear link between the variables

SIGNIFICANCE TEST OF MIDAZOLAM PURE PRODUCT CORRELATION COEFFICIENT (Pearson correlation coefficient test)

R pure product 0,999947635

H0 :	R=0
H1 :	R diffErent from 0
texp	**425,9143808**
Nb of DOF:	19
t threshold (5%, student)	2,093
**Conclusion**	texp > t threshold→ Rejection of H0 at 5% risk, existence of a significant linear link between the variables

Precision

MIDAZOLAM drug product

Essay Number	AUC (mAu*min)	Concentration (µg/mL)	Observed concentration (µg/mL)	Recovery Rate		
D1-1	75,7118	25	23,4240767	94%	Average :	
D1-2	77,5101	25	23,89090771	96%	94%	
D1-3	75,7042	25	23,42210377	94%	Variance:	0,000116723
D2-1	76,7345	25	23,68956526	95%	Average :	
D2-2	80,9352	25	24,78004908	99%	96%	
D2-3	76,7043	25	23,68172547	95%	Variance :	0,000638808
D3-1	79,3289	25	24,36306045	97%	Average :	
D3-2	78,1888	25	24,06709534	96%	97%	
D3-3	79,5651	25	24,42437696	98%	Variance:	5,8401E-05

3,852143345	-14,52110116	Average recovery:	96%

Cochran's C test: study of the homogeneity of a series of variances C (m,p)

m : number of days for 1 test: 6

p: number of tests for validation: 3

s^2 max	0,000638808
sum s^2	8,1393E-04
Cochran's critic C, α = 5%	0,7071
C test	**7,8484E-01**

C test < C 5% : The variances are homogeneous (No rejection of H0)

Estimation of repeatability

Average calculation:	96%
s^2 intra :	2,7131E-04
**SD repeatability:**	0,016821187
	1,68%

Estimation of intermediate precision

Average calculation:	96%
Average s^2 :	8,64965E-08
s^2IG :	5,18979E-07
s^2 factor :	-4,5132E-05
s^2RI:	0,000226179
**SD intermediate precision:**	0,015358509
	1,54%

Precision

MIDAZOLAM within the mixture

Essay Number	AUC (mAu*min)	Concentration (µg/mL)	Observed concentration (µg/mL)	Recovery Rate	
D1-1	98,5854	25	25,15822737	0,993670905	Average :
D1-2	98,7812	25	25,20415971	0,991833612	99%
D1-3	98,7416	25	25,19487002	0,992205199	Variance:
D1-4	97,1472	25	24,82084282	1,007166287	7,457E-05
D1-5	97,9952	25	25,01977349	0,99920906	
D1-6	99,9176	25	25,47074558	0,981170177	
D2-1	98,4846	25	25,13458089	0,994616764	Average :
D2-2	99,4556	25	25,3623659	0,985505364	99%
D2-3	97,9804	25	25,01630159	0,999347936	Variance :
D2-4	98,7816	25	25,20425354	0,991829858	2,8975E-05
D2-5	97,8891	25	24,9948837	1,000204652	
D2-6	98,4027	25	25,11536813	0,995385275	
D3-1	99,7635	25	25,43459556	0,982616178	Average :
D3-2	98,2531	25	25,08027375	0,99678905	99%
D3-3	98,4641	25	25,12977184	0,994809127	Variance:
D3-4	98,0469	25	25,0319017	0,998723932	5,5456E-05
D3-5	98,9687	25	25,24814497	0,990074201	
D3-6	97,4677	25	24,89602829	1,004158868	

4,26279153	-8,658878536	Average recovery:	99%

Cochran's C test: study of the homogeneity of a series of va

m : number of days for 1 test: 6

p: number of tests for validation: 3

s^2 max	7,457E-05
sum s^2	0,000159001
Cochran's critic C, α = 5%	0,7071
C test	**0,468992156**

C test < C 5% : The variances are homogeneous (No rejection of H0)

Estimation of repeatability

Average calculation:	99%
s^2 intra :	5,30002E-05
**SD repeatability:**	0,007300569
	0,73%

Estimation of intermediate precision

Average calculation:	99%
Average s^2 :	5,24253E-10
s^2IG :	3,14552E-09
s^2 factor :	-8,83284E-06
s^2RI:	4,41673E-05
**SD intermediate precision:**	0,006664517
	0,67%

Accuracy

Midazolam drug product

Essay	Observed AUC (mAu*min)	Concentration (mg/L)	observed concentration (mg/L)	Recovery rate (%)	Variance of recoveries	Average recovery	
Day 1 25%	8,1053	5	5,873717339	117,4743468			
Day 2 25%	8,3127	5	5,927557496	118,5511499			
Day 3 25%	7,0145	5	5,590550307	111,8110061	**13,11041174**	115,9455009	
Day 1 50%	9,6464	6	6,273780333	104,5630056			
Day 2 50%	10,0161	6	6,369752878	106,162548			
Day 3 50%	10,499	6	6,495111661	108,251861	**3,421903252**	106,3258048	
Day 1 100%	15,5306	8	7,801293583	97,51616979			
Day 2 100%	16,4835	8	8,048662363	100,6082795			
Day 3 100%	16,3314	8	8,009177851	100,1147231	**2,759536692**	99,41305749	
Day 1 125%	19,9329	10	8,944111907	89,44111907			
Day2 125%	21,3962	10	9,323978352	93,23978352			
Day 3 125%	24,1843	10	10,04775723	100,4775723	**31,43645653**	94,38615831	
Day 1 150%	59,5402	20	19,22599824	96,12999118			
Day 2 150%	63,6142	20	20,28359128	101,4179564			
Day 3 150%	61,0744	20	19,62427002	98,1213501	**7,132616581**		
Day 1 200%	77,2558	25	23,82489252	95,29957007			**5,245211878**
Day 2 200%	79,6734	25	24,45249118	97,80996471			
Day 3 200%	79,4095	25	24,38398386	97,53593545	**1,896417229**	96,88182341	
Day 1 250%	177,0261	50	49,72483732	99,44967464			
Day 2 250%	184,5178	50	51,66965072	103,3393014			
Day 3 250%	178,4899	50	50,10483356	100,2096671	**4,250232698**	100,9995477	
				Sum of variances	64,00757473	99,60127836	Average of recovery averages
						2,745211878	Total standard deviation of the recovery
	3,852143345	-14,52110116					

Cochran's C test C(7,3)

C test	**0,491136505**
C threshold (cochran table) 5%	0,561
s^2max	31,43645653
s^2 sum	64,00757473

C test < C table, no rejection of H0, the variances are homogeneous at the 5% risk.

ANOVA

	Sum of SES	DOF	s^2	Fexp	p-value
Intragroup	128,0151495	14	9,143939247		
Intergroup	949,7680906	5	189,9536181	20,77371831	5,03737E-06
Total	1077,78324	19			

SES : Sum of standard errors squared

Mean Recovery Confidence Interval

**Average R:**	101,7869036
**total variance :**	56,72543369
**Number of DOF:**	n-1 =20
**t 5%, 20 DOF:**	2,085963447
**lower bound :**	97,73041766
**Upper bound :**	105,8433896

So the mean overlap confidence interval is : [97,7304 ; 105,8434]

The average recovery rate is 101,7869

It is included in the confidence interval: ok.

Accuracy

Midazolam within the mixture

Essay	Observed AUC (mAu*min)	Concentration (mg/L)	observed concentration (mg/L)	Recovery rate (%)	Variance of recoveries	Average recovery	
Day 1 25%	11,9606	5	4,837083491	96,74166982			
Day 2 25%	12,5097	5	4,965895795	99,3179159			
Day 3 25%	12,3668	5	4,932373161	98,64746323	**1,786433202**	98,23568298	
Day 1 50%	16,7904	6	5,970096908	99,50161514			
Day 2 50%	16,1378	6	5,81700474	96,950079			
Day 3 50%	16,8438	6	5,982623911	99,71039851	**2,362215174**	98,72069755	
Day 1 100%	25,0072	8	7,897660089	98,72075111			
Day 2 100%	26,1641	8	8,169055017	102,1131877			
Day 3 100%	25,109	8	7,921541154	99,01926442	**3,5283496**	99,95106775	
Day 1 125%	34,2853	10	10,07419158	100,7419158			
Day2 125%	34,1191	10	10,03520304	100,3520304			
Day 3 125%	34,4575	10	10,11458764	101,1458764	**0,157564377**	100,7466075	
Day 1 150%	75,9421	20	19,84637952	99,23189761			
Day 2 150%	76,9176	20	20,07522018	100,3761009			
Day 3 150%	76,0807	20	19,87889343	99,39446714	**0,383205795**		
Day 1 200%	99,4188	25	25,35373306	101,4149322			
Day 2 200%	98,0661	25	25,03640579	100,1456231			
Day 3 200%	99,5831	25	25,39227588	101,5691035	**0,610201774**	101,0432196	
Day 1 250%	204,1925	50	49,9323922	99,86478439			
Day 2 250%	204,0261	50	49,89335674	99,78671348			
Day 3 250%	203,9437	50	49,87402669	99,74805337	**0,003535967**	99,79985042	
				Sum of variances	8,831505889	99,74952098	Average of recovery averages
						1,33910121	Total standard deviation of the recovery
	4,26279153	-8,658878536					

Cochran's C test C(7,3)

C test	**0,399518456**
C threshold (cochran	0,561
s^2max	3,5283496
s^2 sum	8,831505889

C test < C table, no rejection of H0, the variances are homogeneous at the 5% risk.

ANOVA

	Sum of SES	DOF	s^2	Fexp	p-value
Intragroup	17,66301178	14	1,261643698		
Intergroup	18,20082925	5	3,640165851	2,885256634	0,053900355
Total	35,86384103	19			

Conditions du test d'ANOVA:
- Homogénéité des variances
- Normalité des résidus
- Indépendance des données

SES : Sum of standard errors squared

Mean Recovery Confidence Interval

**Average R:**	99,73780206
**total variance :**	1,887570581
**Number of DOF:**	n-1 =20
**t 5%, 20 DOF:**	2,085963447
**lower bound :**	98,99783453
**Upper bound :**	100,4777696

So the mean overlap confidence interval is: [98,9978 ; 100,4778]

The average recovery rate is 99,7378

It is included in the confidence interval: ok.
