# Supplementary material for: Therapeutic Treatment Plan Optimization during the COVID-19 Pandemic: A Comprehensive Physicochemical Compatibility Study of Intensive Care Units Selected Drugs
Source: Pharmaceutics. 2022 Feb 28;14(3):550. doi: 10.3390/pharmaceutics14030550 (PMC8952813; doi:10.3390/pharmaceutics14030550)
Supplement: Supplementary file 1 [file pharmaceutics-14-00550-s001.zip › Supplementary materials Figure S1E-Sufentanil method Validation.pdf]

Linearity

SUFENTANIL drug product

Essay	Observed AUC (mAu)	Concentration (µg/L)	Variance s²	Estimated AUC	Adjusted AUC	Confidence interval
70% D1	29,9176	0,7	0,113118493	29,77485119	29,9176	0,229153626
70% D2	29,397	0,7		29,77485119	29,397	-0,606561768
70% D3	29,2884	0,7		29,77485119	29,2884	-0,780896558
80% D1	32,3645	0,8	0,76291591	31,21847381	32,3645	1,839707508
80% D2	30,6921	0,8		31,21847381	30,6921	-0,844984048
80% D3	31,0912	0,8		31,21847381	31,0912	-0,204311721
90% D1	33,5192	0,9	0,494723703	32,66209643	33,5192	1,375902129
90% D2	32,3083	0,9		32,66209643	32,3083	-0,567946833
90% D3	32,2937	0,9		32,66209643	32,2937	-0,591384107
100% D1	34,1135	1	0,010401943	34,10571905	34,1135	0,012490706
100% D2	34,2246	1		34,10571905	34,2246	0,190838728
100% D3	34,3172	1		34,10571905	34,3172	0,339488835
110% D1	36,1929	1,1	0,281481943	35,54934167	36,1929	1,033099512
110% D2	35,5591	1,1		35,54934167	35,5591	0,015664982
110% D3	35,139	1,1		35,54934167	35,139	-0,658718493
120% D1	38,0203	1,2	1,029620653	36,99296429	38,0203	1,64917455
120% D2	36,7763	1,2		36,99296429	36,7763	-0,347809602
120% D3	36,0097	1,2		36,99296429	36,0097	-1,578427006
130% D1	39,3044	1,3	0,813711143	38,4365869	39,3044	1,39309405
130% D2	38,1685	1,3		38,4365869	38,1685	-0,430357958
130% D3	37,5226	1,3		38,4365869	37,5226	-1,46721653

Linear regression (LINEST function):

Slope b	14,43622619	19,66949286	Intercept a
S slope	0,679682548	0,693142915	S Intercept
R²	0,959585058	0,622939345	S res
F	451,1231542	19	DOF
Regression sum of squares	175,0598864	7,373015129	Residual sum of squares

CHECKING THE FIT TO THE LINEAR MODEL n=3 k=7

Cochran's C test: Study of the homogeneity of a series of variances

C test	**0,293676084**	*k : number of samples per test*	
Cochran's critical C, α = 5%	0,561	*C test = max s²/sum s²*	
Maximum S²	1,029620653		0,293676084
Sum s²	3,50597379		

The variances are homogeneous at the risk of 0.05% because 0.294<0.561

Calculation of experimental variance s²exp

s²exp = 0,500853399

Calculation of misfit variance

SCE aj = 2,364481144

s² aj = 0,788160381

Fisher Test

H0: Not significantly different

H1: Significantly different

Fexp 1,573634887 **F seuil 5% (5, 14)** 2,958248913 **p-value** 0,231059692

Conclusion p-value > 5%, therefore no rejection of H0: Fit to the validated linear model.

DEPENDENCE VERIFICATION. F(1, n - 2)

H0: The variances are not significantly different

H1: The variances are significantly different

F exp **451,1231542** **F threshold 5% (1, 19)** 4,38 **p-value** 1,06437E-14

Conclusion Dependence is validated as H0 is rejeted, p-value<5%

Linearity

SUFENTANIL within the mixture

Essay	Observed AUC (mAu)	Concentration (µg/L)	Variance s²	Estimated AUC	Adjusted AUC	Confidence interval
70% D1	32,5024	0,7	0,19813996	33,13350595	32,5024	-1,525704847
70% D2	33,047	0,7		33,13350595	33,047	-0,209128991
70% D3	33,3846	0,7		33,13350595	33,3846	0,60702233
80% D1	34,4409	0,8	0,266438013	34,57480238	34,4409	-0,323710323
80% D2	34,1067	0,8		34,57480238	34,1067	-1,13164211
80% D3	35,1197	0,8		34,57480238	35,1197	1,317295354
90% D1	36,0963	0,9	0,259439053	36,01609881	36,0963	0,193887167
90% D2	35,2057	0,9		36,01609881	35,2057	-1,959147093
90% D3	36,0793	0,9		36,01609881	36,0793	0,1527895
100% D1	38,2199	1	0,08411863	37,45739524	36,77860357	1,843362762
100% D2	37,8916	1		37,45739524	37,8916	1,049694283
100% D3	37,6416	1		37,45739524	37,6416	0,445316824
110% D1	39,2214	1,1	0,051236943	38,89869167	39,2214	0,78015057
110% D2	39,3788	1,1		38,89869167	39,3788	1,160666618
110% D3	38,9325	1,1		38,89869167	38,9325	0,081731978
120% D1	40,4748	1,2	0,025822813	40,3399881	40,4748	0,325909106
120% D2	40,1538	1,2		40,3399881	40,1538	-0,450111552
120% D3	40,328	1,2		40,3399881	40,328	-0,028981338
130% D1	41,4144	1,3	0,03945499	41,78128452	41,4144	-0,886946945
130% D2	41,6776	1,3		41,78128452	41,6776	-0,250658356
130% D3	41,2883	1,3		41,78128452	41,2883	-1,191794935

Linear regression (LINEST function):

Slope b	14,41296429	23,04443095	Intercept a
S slope	0,451327823	0,460265875	S Intercept
R²	0,981709971	0,413648782	S res
F	1019,817374	19	DOF
Regression sum of squares	174,4961732	3,251000988	Residual sum of squares

CHECKING THE FIT TO THE LINEAR MODEL n=3 k=7

Cochran's C test: Study of the homogeneity of a series of variances

C test	0,28815
Cochran's critical C, α = 5%	0,561
Maximum S²	0,266438013
Sum s²	0,924650403

The variances are homogeneous at the risk of 0.05% because 0.288<0.561

Calculation of experimental variance s²exp

s²exp = 0,132092915

Calculation of misfit variance

SCE aj = 1,930071841

s² aj = 0,64335728

Fisher Test

H0: Not significantly different

H1: Significantly different

Fexp 4,870490453 **F seuil 5% (5, 14)** 2,958248913 p-value 0,008645393

Conclusion p-value > 5%, therefore no rejection of H0: Fit to the validated linear model.

DEPENDENCE VERIFICATION. F(1, n - 2)

H0: The variances are not significantly different

H1: The variances are significantly different

F exp **1019,817374** **F threshold 5% (1, 19)** 4,38 **p-value** 5,64303E-18

Conclusion Dependence is validated as H0 is rejeted, p-value<5%

VALIDATION OF THE PROPORTIONAL MODEL

Regression of SUFENTANIL within the mixture: Comparison of the y-intercept to 0

Ial	19,66949286
Standard deviation of the intercept	0,693142915

Student Test

**H0:**	The y-intercept is equal to 0
**H1:**	The y-intercept is different from 0
**t exp**	**28,37725442**
**Nb of DOF (N-2)**	19
**t threshold (5%, student)**	2,093

Conclusion texp > t threshold → rejet of H0 at 5% risk. The y-intercept is significantly different from 0

the correlation line doesn't pass through the origin

SUFENTANIL Pure drug Product Regression: Comparing the Intercept to 0

Ial	23,04443095
Standard deviation	0,460265875

Student Test

**H0:**	The y-intercept is equal to 0
**H1:**	The y-intercept is different from 0
**t exp**	**50,06765044**
**Nb of DOF (N-2)**	19
**t threshold (5%, student)**	2,093

Conclusion texp > t threshold → rejet of H0 at 5% risk. The y-intercept is significantly different from 0

the correlation line doesn't pass through the origin

CALCULATED LIMIT OF DETECTION	**0,431511211**
CALCULATED LIMIT OF QUANTIFICATION	**0,1423987**

COMPARISON OF CLONIDINE IN THE MIXTURE AND THE PUR DRUG PRODUCT REGRESSIONS

Comparison of Student slopes

**H0:**	No difference between the two slopes
**H1:**	Difference between the two slopes
**texp**	**0,028511322**
**Nb of DOF (N1+N2-4)**	38
**t threshold (5%, student à 38 dof)**	2,042

Conclusion texp < t threshold → No rejection of H0 at 5% risk, no significant difference between the 2 slopes.

COMPARISON OF STUDENT INTERCEPTS

**H0:**	No difference between the two intercepts
**H1:**	Difference between the two intercepts
**t exp**	**4,056217408**
**Nb of DOF (N1+N2-4)**	38
**t threshold (5%, student à 38 ddl)**	2,042

Conclusion texp > t threshold→ rejection of H0 at 5% risk, therefore systematic error.

SIGNIFICANCE TEST OF SUFENTANIL IN THE MIXTURE FORM CORRELATION COEFFICIENT (Pearson correlation coefficient test)

R reconstituted form: 5,684039057

**H0:**	R=0
**H1:**	R different from 0
**texp**	**123,2431739**
**Nb of DOF**	19
**t threshold (5%, student)**	2,093

Conclusion texp > t threshold→ Rejection of H0 at 5% risk, existence of a significant linear link between the variables

SIGNIFICANCE TEST OF SUFENTANIL PURE PRODUCT CORRELATION COEFFICIENT (Pearson correlation coefficient test)

R pure product 0,990812783

**H0 :**	R=0
**H1 :**	R different from 0
**texp**	**31,93457959**
**Nb of DOF:**	19
**t threshold (5%, student)**	2,093

Conclusion texp > t threshold→ Rejection of H0 at 5% risk, existence of a significant linear link between the variables

Precision

SUFENTANIL drug product

Essay Number	AUC (mAu*min)	Concentration (µg/mL)	Observed concentration (µg/mL)	Recovery Rate	
Day 1-1	34,5353	1	1,02975715	0,97024285	Average:
Day 1-2	34,0439	1	0,995717783	1,004282217	102%
Day 1-3	33,9958	1	0,992385888	1,007614112	Variance:
Day 1-4	33,3699	1	0,949029681	1,050970319	0,000976774
Day 1-5	33,4586	1	0,955173946	1,044826054	
Day 1-6	33,5168	1	0,959205471	1,040794529	
Day 2-1	33,4472	1	0,954384266	1,045615734	Average :
Day 2-2	33,3014	1	0,944284674	1,055715326	104%
Day 2-3	33,4063	1	0,951551116	1,048448884	Variance :
Day 2-4	33,9534	1	0,989448832	1,010551168	0,000292599
Day 2-5	33,5224	1	0,959593384	1,040406616	
Day 2-6	33,2782	1	0,942677606	1,057322394	
Day 3-1	33,5635	1	0,962440388	1,037559612	Average:
Day 3-2	34,1702	1	1,004466607	0,995533393	100%
Day 3-3	34,2548	1	1,010326865	0,989673135	Variance:
Day 3-4	33,8978	1	0,98559741	1,01440259	0,00039867
Day 3-5	34,1569	1	1,003545314	0,996454686	
Day 3-6	34,3486	1	1,016824408	0,983175592	
	14,43622619	19,66949286	**Average recovery:**	102%	

Cochran's C test: study of the homogeneity of a series of variances C (m,p)

m : number of days for 1 test: 6

p: number of tests for validation: 3

s^2 max	0,000976774
sum s^2	0,001668043
Cochran's critique C, α = 5%	0,7071
C test	**0,585580916**
C test < C 5% :	The variances are homogeneous (No rejection of H0)

Estimation of repeatability

Average calculation:	102%
s^2 intra :	0,000556014
**SD repeatability:**	0,023326307
	2,33%

Estimation of intermediate precision

Average calculation:	102%
Average s^2 :	1,35592E-07
s^2IG :	8,13551E-07
s^2 facteur :	-9,25335E-05
s^2RI:	0,000463481
**SD intermediate precision:**	0,021297023
	2,13%

Precision

SUFENTANIL within the mixture

Essay Number	AUC (mAu*min)	Concentration (µg/mL)	Observed concentration (µg/mL)	Recovery Rate	
Day 1-1	37,3353	1	0,991528791	1,008471209	Average:
Day 1-2	37,0607	1	0,972476499	1,027523501	105%
Day 1-3	36,6511	1	0,94405764	1,05594236	Variance:
Day 1-4	35,937	1	0,894511968	1,105488032	0,001182606
Day 1-5	36,7745	1	0,952619376	1,047380624	
Day 1-6	36,3756	1	0,924942904	1,075057096	
Day 2-1	37,0242	1	0,969944057	1,030055943	Average :
Day 2-2	37,3203	1	0,990488061	1,009511939	102%
Day 2-3	38,2879	1	1,057622065	0,942377935	Variance :
Day 2-4	36,7447	1	0,950551793	1,049448207	0,002313497
Day 2-5	37,5301	1	1,0050444	0,9949556	
Day 2-6	36,2738	1	0,917879819	1,082120181	
Day 3-1	38,2014	1	1,051620524	0,948379476	Average:
Day 3-2	37,9783	1	1,036141404	0,963858596	96%
Day 3-3	37,6075	1	1,010414566	0,989585434	Variance:
Day 3-4	37,6624	1	1,014223636	0,985776364	0,002144187
Day 3-5	39,1903	1	1,120232364	0,879767636	
Day 3-6	37,29147647	1	0,988488227	1,011511773	
	14,41296429	23,04443095	**Average recovery:**	101%	

Cochran's C test: study of the homogeneity of a series of variances

m : number of days for 1 test: 6

p: number of tests for validation: 3

s^2 max	0,002313497
sum s^2	0,00564029
Cochran's critique C, α = 5%	0,7071
C test	**0,410173401**
C test < C 5% :	The variances are homogeneous (No rejection of H0)

Estimation of repeatability

Average calculation:	101%
s^2 intra :	0,001880097
**SD repeatability:**	0,04311264
	4,31%

Estimation of intermediate precision

Average calculation:	101%
Average s^2 :	3,72036E-07
s^2IG :	2,23222E-06
s^2 facteur :	-0,000312977
s^2RI:	0,001567119
**SD intermediate precision:**	0,039360948
	3,94%

Accuracy

SUFENTANIL drug product

Essay	Observed AUC (mAu*min)	Concentration (mg/L)	observed concentration (mg/L)	Recovery rate (%)	Variance of recoveries	Average recovery	
**Day 1 25%**	29,9176	0,7	0,709888236	101,4126051	11,07720456	97,61989779	
**Day 2 25%**	29,397	0,7	0,67382618	96,26088281			
**Day 3 25%**	29,2884	0,7	0,666303438	95,18620543			
**Day 1 50%**	32,3645	0,8	0,879385442	109,9231802	57,19911927	101,4211314	
**Day 2 50%**	30,6921	0,8	0,763537991	95,44224887			
**Day 3 50%**	31,0912	0,8	0,79118372	98,89796502			
**Day 1 100%**	33,5192	0,9	0,959371719	106,5968577	29,30693114	100,3461218	
**Day 2 100%**	32,3083	0,9	0,875492457	97,27693971			
**Day 3 100%**	32,2937	0,9	0,874481113	97,16456808			
**Day 1 125%**	34,1135	1	1,000538988	100,0538988	0,499122476	100,7807739	
**Day2 125%**	34,2246	1	1,008234905	100,8234905			
**Day 3 125%**	34,3172	1	1,014649324	101,4649324			
**Day 1 150%**	36,1929	1,1	1,144579402	104,0526729	11,16240599	100,5100279	
**Day 2 150%**	35,5591	1,1	1,100675962	100,061451			
**Day 3 150%**	35,139	1,1	1,071575559	97,41595986			
**Day 1 200%**	38,0203	1,2	1,271163731	105,9303109	34,30894766	99,66790171	
**Day 2 200%**	36,7763	1,2	1,184991626	98,74930214			
**Day 3 200%**	36,0097	1,2	1,131889105	94,32409209			
**Day 1 250%**	39,3044	1,3	1,36011357	104,6241208	23,10341625	99,44182317	
**Day 2 250%**	38,1685	1,3	1,281429572	98,57150551			
**Day 3 250%**	37,5226	1,3	1,236687962	95,12984321			
				Sum of variances	166,6571473	99,96966824	Average of recovery averages
						4,343152317	Total standard deviation of the recovery

14,43622619 19,66949286

Cochran's C test C(7,3)

C test	**0,343214319**
C threshold (cochran	0,561
s^2max	57,19911927
s^2 sum	166,6571473

C test < C table, no rejection of H0, the variances are homogeneous at the 5% risk.

ANOVA

	Sum of SES	DOF	s^2	Fexp	p-value
**Intragroup**	333,3142947	14	23,8081639		
**Intergroup**	27,26834442	5	5,453668885	0,229067177	0,943563845
**Total**	360,5826391	19			

SES : Sum of standard errors squared

Mean Recovery Confidence Interval

**Average R:**	99,96966824
**total variance :**	18,97803364
**Number of DOF:**	n-1 =20
**t 5%, 20 DOF:**	2,085963447
**lower bound :**	97,62335133
**Upper bound :**	102,3159852

So the mean overlap confidence interval is : [99,1487 ; 101,9262]

The average recovery rate is 100,5374

It is included in the confidence interval: ok.

Accuracy

SUFENTANIL within the mixture

Essay	Observed AUC (mAu*min)	Concentration (mg/L)	observed concentration (mg/L)	Recovery rate (%)	Variance of recoveries	Average recovery	
**Day 1 25%**	32,5024	0,7	0,656212619	93,74465992	19,4656706	98,45866987	
**Day 2 25%**	33,047	0,7	0,693998046	99,14257796			
**Day 3 25%**	33,3846	0,7	0,717421402	102,4887717			
**Day 1 50%**	34,4409	0,8	0,790709588	98,83869846	20,04054795	99,83490806	
**Day 2 50%**	34,1067	0,8	0,76752213	95,94026625			
**Day 3 50%**	35,1197	0,8	0,837806076	104,7257595			
**Day 1 100%**	36,0963	0,9	0,905564517	100,6182797	15,41855631	98,2860173	
**Day 2 100%**	35,2057	0,9	0,843772926	93,75254733			
**Day 3 100%**	36,0793	0,9	0,904385024	100,4872249			
**Day 1 125%**	38,2199	1	1,05290409	105,290409	4,049352368	103,1936856	
**Day2 125%**	37,8916	1	1,030125986	103,0125986			
**Day 3 125%**	37,6416	1	1,012780491	101,2780491			
**Day 1 150%**	39,2214	1,1	1,122390143	102,0354676	2,0384085	101,7589909	
**Day 2 150%**	39,3788	1,1	1,133310867	103,0282606			
**Day 3 150%**	38,9325	1,1	1,102345689	100,2132445			
**Day 1 200%**	40,4748	1,2	1,209353517	100,7794597	0,863245738	99,87787946	
**Day 2 200%**	40,1538	1,2	1,187081901	98,92349177			
**Day 3 200%**	40,328	1,2	1,199168242	99,93068685			
**Day 1 250%**	41,4144	1,3	1,274544825	98,04190964	1,123850678	98,28581398	
**Day 2 250%**	41,6776	1,3	1,292806162	99,44662788			
**Day 3 250%**	41,2883	1,3	1,265795758	97,36890443			
				Sum of variances	62,99963214	99,95656645	Average of recovery averages
						3,088064046	Total standard deviation of the recovery

14,41296429 23,04443095

Cochran's C test C(7,3)

C test	**0,318105793**
C threshold (cochran tab	0,561
s^2max	20,04054795
s^2 sum	62,99963214

C test < C table, no rejection of H0, the variances are homogeneous at the 5% risk.

ANOVA

	Sum of SES	DOF	s^2	Fexp	p-value
**Intragroup**	125,9992643	14	8,999947449		
**Intergroup**	64,72352671	5	12,94470534	1,438308992	0,270926903
**Total**	190,722791	19			

SES : Sum of standard errors squared

Mean Recovery Confidence Interval

**Average R:**	99,95656645
**total variance :**	10,03804163
**Number of DOF:**	n-1 =20
**t 5%, 20 DOF:**	2,085963447
**lower bound :**	98,25014791
**Upper bound :**	101,662985

So the mean overlap confidence interval is : [99,1487 ; 101,9262]

The average recovery rate is 100,5374

It is included in the confidence interval: ok.
