# Supplementary material for: Therapeutic Treatment Plan Optimization during the COVID-19 Pandemic: A Comprehensive Physicochemical Compatibility Study of Intensive Care Units Selected Drugs
Source: Pharmaceutics. 2022 Feb 28;14(3):550. doi: 10.3390/pharmaceutics14030550 (PMC8952813; doi:10.3390/pharmaceutics14030550)
Supplement: Supplementary file 1 [file pharmaceutics-14-00550-s001.zip › Supplementary materials Figure S2.pdf]

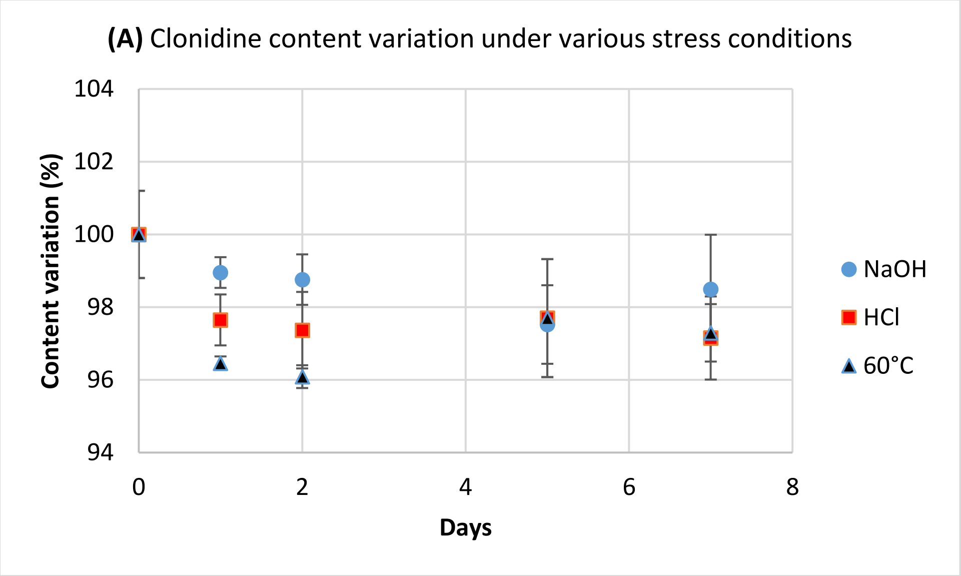
(A) Clonidine content variation under various stress conditions
104
102
100
98
96
94
Content variation (%)
0
2
4
6
8
Days
NaOH
HCl
60°C

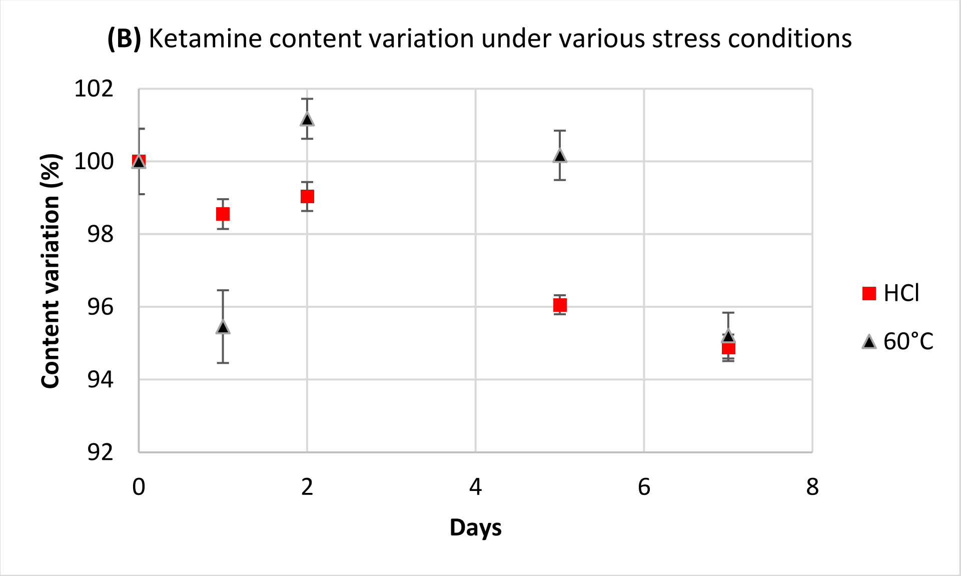
(B) Ketamine content variation under various stress conditions
102
100
98
96
94
92
Content variation (%)
0
2
4
6
8
Days
HCl
60°C

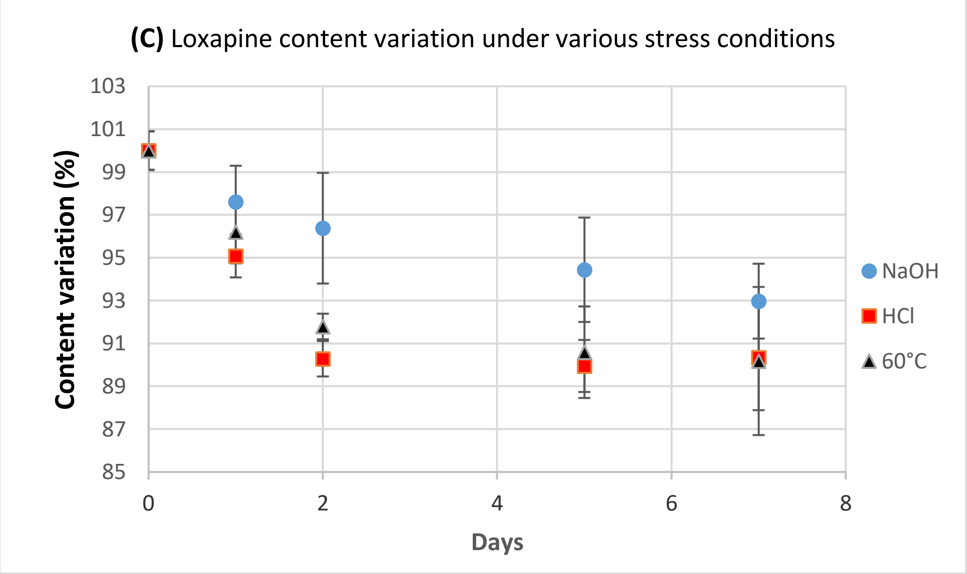
(C) Loxapine content variation under various stress conditions
103
101
99
97
95
93
91
89
87
85
Content variation (%)
0
2
4
6
8
Days
NaOH
HCl
60°C

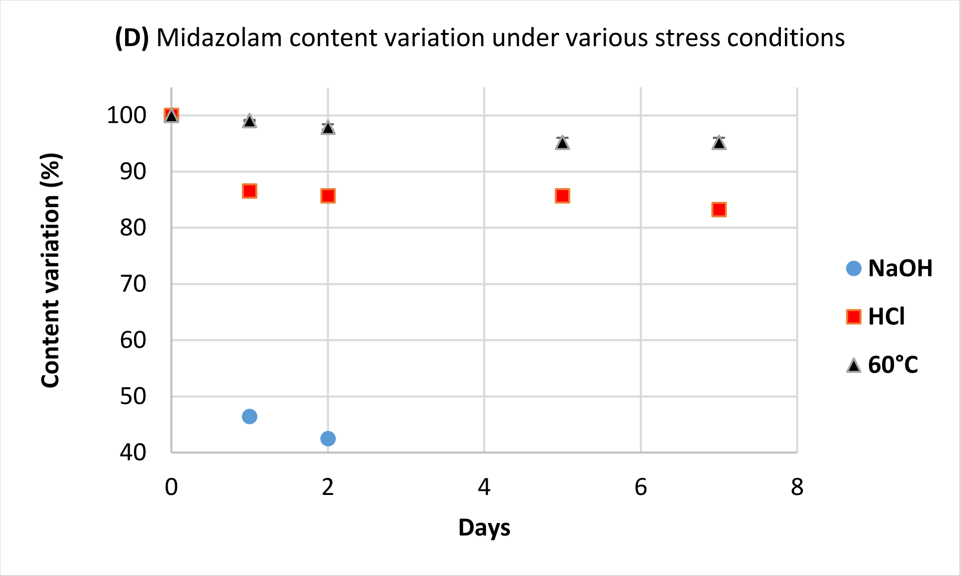

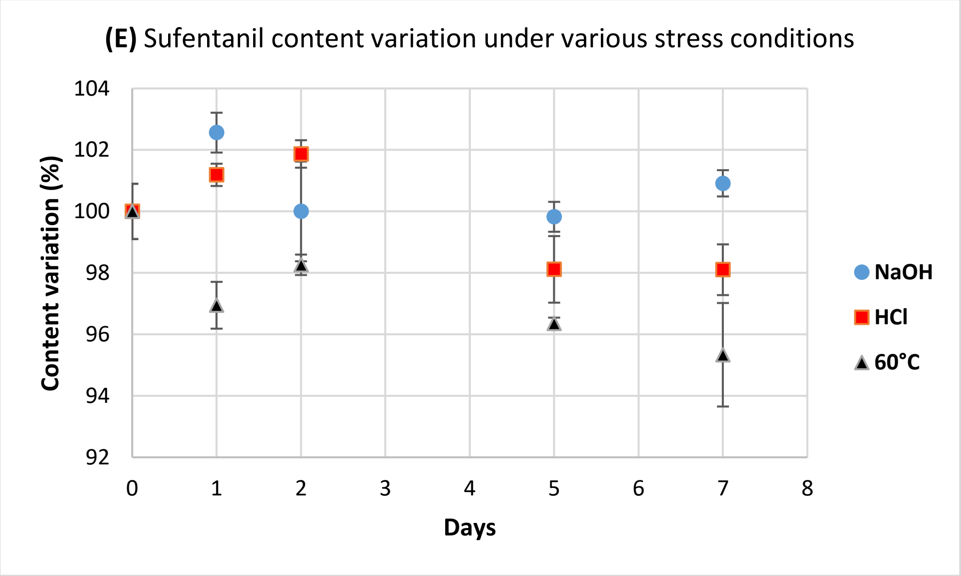

Supplementary materials Figure S2: Observed content variation under stress conditions (as mean percentage of initial concentration±SD) for each drug: A) Clonidine; B) Ketamine; C) Loxapine; D) Midazolam; E) Sufentanil. The red square dots show HCl 1N stress condition at 25 °C; the rounded blue dots indicate NaOH 1N stress condition at 25 °C; and the triangular black dots indicate thermal stress condition at 60 °C.
