# Supplementary material for: Therapeutic Treatment Plan Optimization during the COVID-19 Pandemic: A Comprehensive Physicochemical Compatibility Study of Intensive Care Units Selected Drugs
Source: Pharmaceutics. 2022 Feb 28;14(3):550. doi: 10.3390/pharmaceutics14030550 (PMC8952813; doi:10.3390/pharmaceutics14030550)
Supplement: Supplementary file 1 [file pharmaceutics-14-00550-s001.zip › Supplementary materials Figure S3A NaCl.pdf]

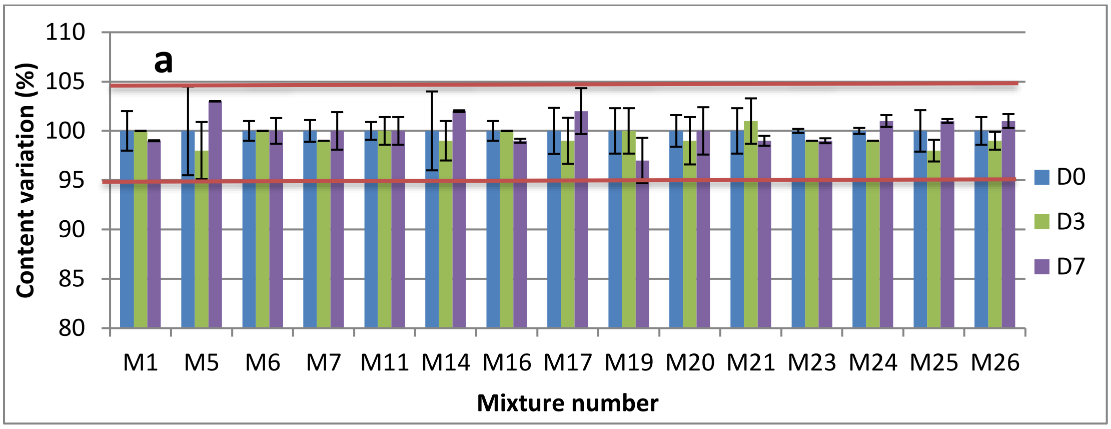
a
Content variation (%)
110
105
100
95
90
85
80
M1 M5 M6 M7 M11 M14 M16 M17 M19 M20 M21 M23 M24 M25 M26
Mixture number
D0
D3
D7

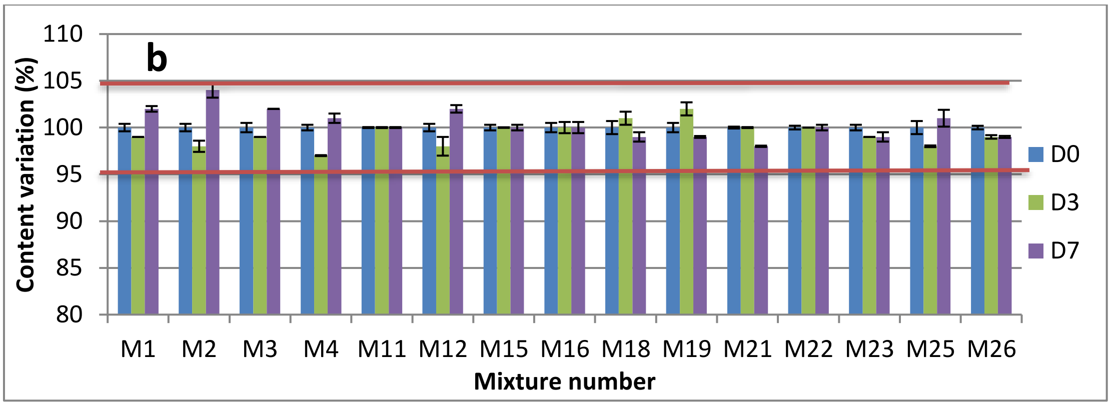
b
Content variation (%)
110
105
100
95
90
85
80
M1 M2 M3 M4 M11 M12 M15 M16 M18 M19 M21 M22 M23 M25 M26
Mixture number
D0
D3
D7

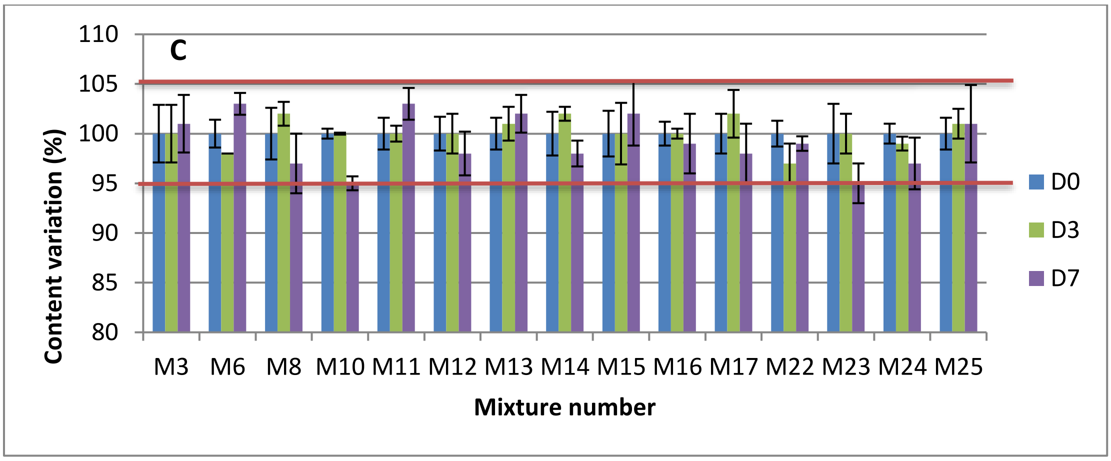
c
Content variation (%)
110
105
100
95
90
85
80
M3 M6 M8 M10 M11 M12 M13 M14 M15 M16 M17 M22 M23 M24 M25
Mixture number
D0
D3
D7

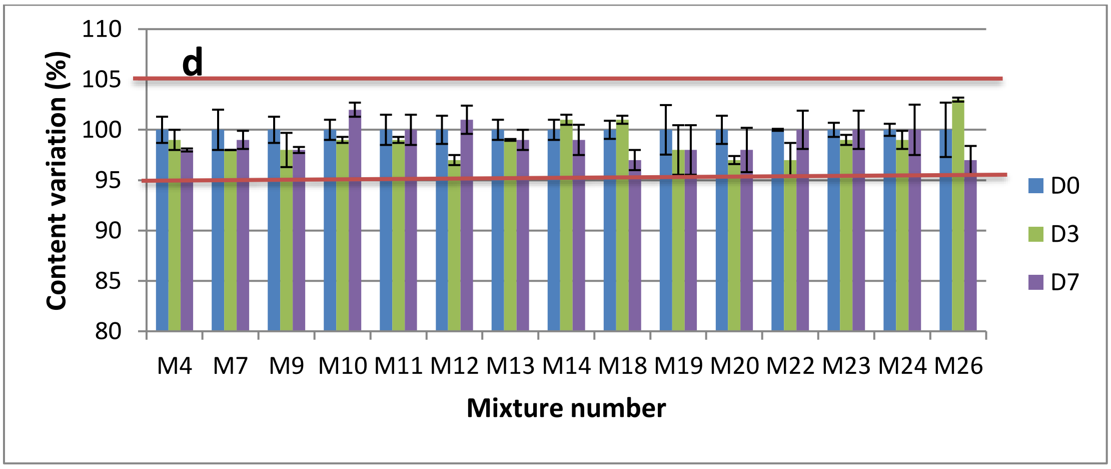
d
Content variation (%)
110
105
100
95
90
85
80
M4 M7 M9 M10 M11 M12 M13 M14 M18 M19 M20 M22 M23 M24 M26
Mixture number
D0
D3
D7

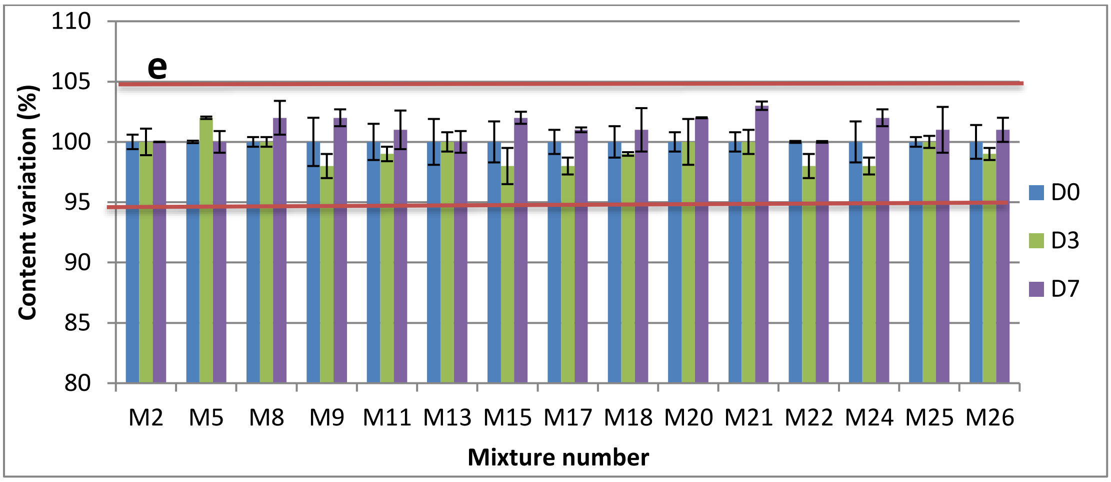

Figure S3A - Drugs mixture diluted in NaCl: a) Clonidine; b) Sufentanil; c) Loxapine; d) Midazolam; e) Ketamine
