# Supplementary figures and images for: Therapeutic Treatment Plan Optimization during the COVID-19 Pandemic: A Comprehensive Physicochemical Compatibility Study of Intensive Care Units Selected Drugs
Source: Pharmaceutics. 2022 Feb 28;14(3):550. doi: 10.3390/pharmaceutics14030550 (PMC8952813; doi:10.3390/pharmaceutics14030550)

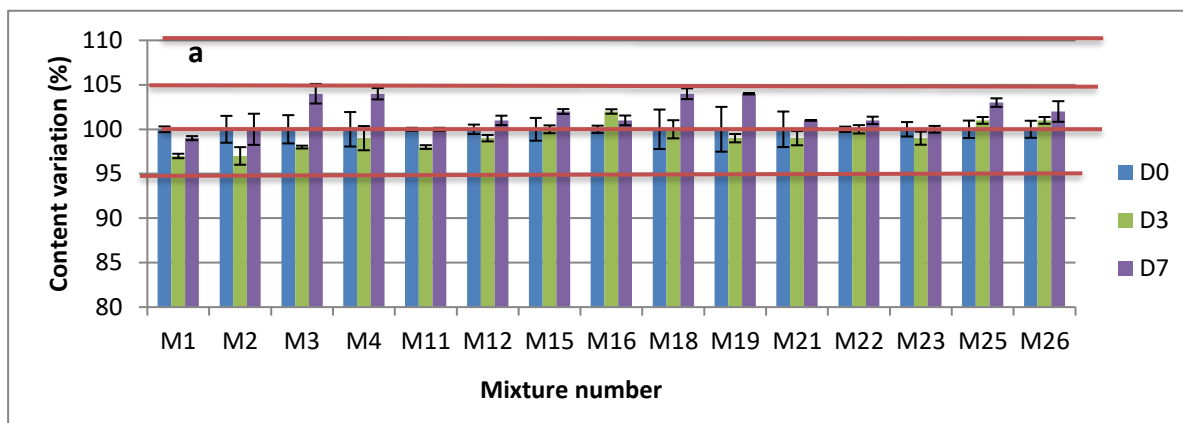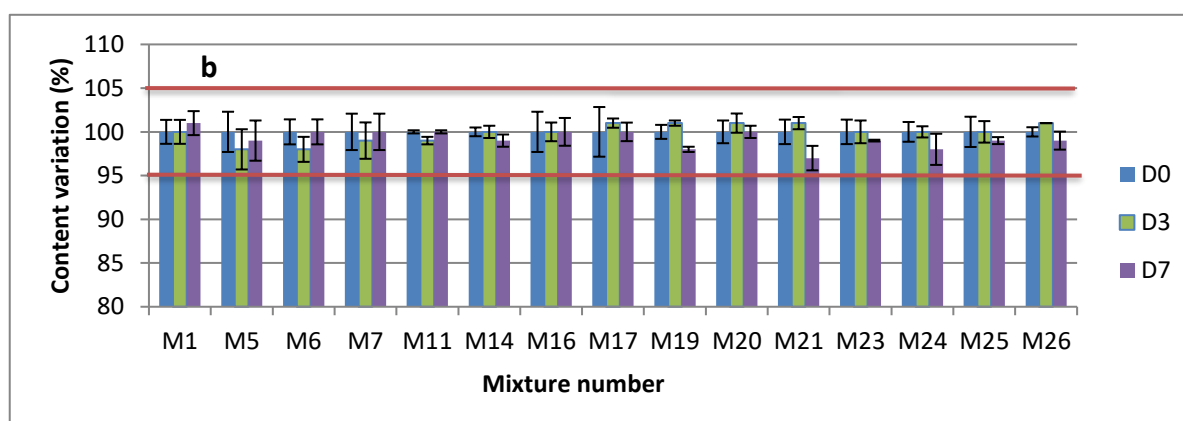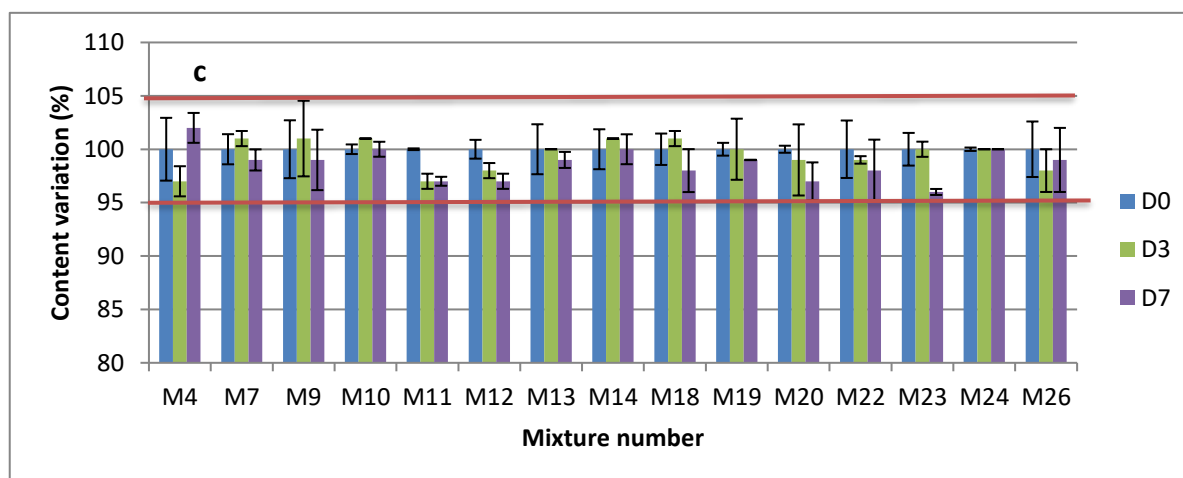

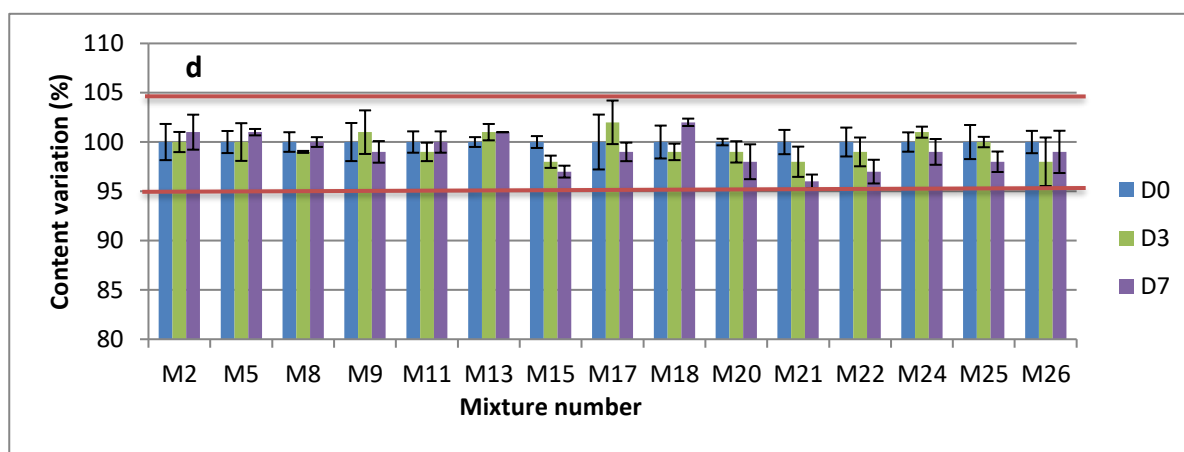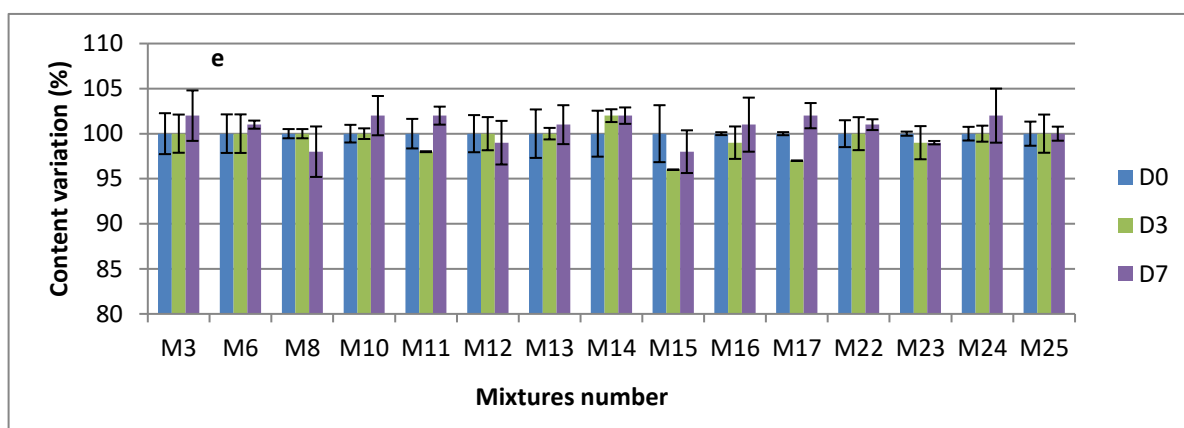

**Figure S3B** - Drugs mixture diluted in G5%: a) Sufentanil ; b) Clonidine ; c) Midazolam ; d) Ketamine; e) Loxapine

Supplement: Supplementary file 1 [file pharmaceutics-14-00550-s001.zip › Supplementary materials Figure S3B G5%.pdf]

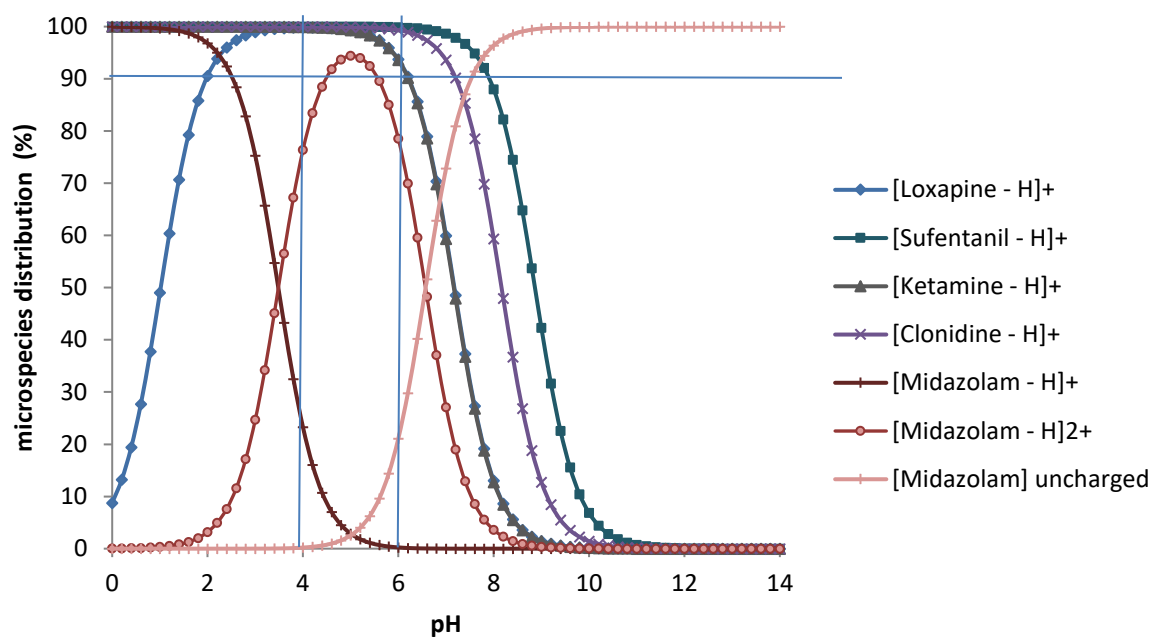

**Figure S4.** Microspecies distribution of the 5 drugs individually according to the pH

Supplement: Supplementary file 1 [file pharmaceutics-14-00550-s001.zip › Supplementary materials Figure S4 Microspecies distribution of the 5 drugs individually according to the pH.pdf]
